# A different world: temporal changes in the community structure of sea slugs (Heterobranchia) in northwest Japan spanning more than a half-century

**DOI:** 10.7717/peerj.20870

**Published:** 2026-03-02

**Authors:** Riko Kato, Mitsuharu Yagi

**Affiliations:** Graduate School of Fisheries and Environmental Sciences, Nagasaki University, Nagasaki, Japan

**Keywords:** Biological diversity, Climate change, SCUBA, Heterobranchia, Opisthobanchia

## Abstract

Understanding long-term changes in marine biodiversity is essential for evaluating effects of climate change on coastal ecosystems. In this study, we compared heterobranch sea slug (hereafter referred to simply as ‘sea slugs’) assemblages in northwestern Kyushu, Japan, across three survey periods: (1) historical records from 1960–1980, for which the exact survey effort and duration were not documented; (2) surveys conducted from April 2001 to September 2003; and (3) recent surveys conducted from June 2023 to January 2024. A total of 47 sea slug species were recorded from our underwater surveys conducted in 2023–2024. Species diversity indices, including the Shannon–Wiener diversity index (*H*) and Simpson’s diversity index (*D*), showed higher values during this survey than those of 2001–2003. Comparative analysis of species composition revealed significant shifts, with 15 species exhibiting statistically significant differences in relative abundance from the past to the present. Notably, several species common in the past, such as Aplysia kurodai, were rarely observed in the recent survey, while many tropical–subtropical species appeared for the first time. The proportion of tropical–subtropical species increased markedly, whereas subarctic species were no longer detected. Jaccard’s coefficient indicated that the current community differs markedly from those in earlier periods. These findings suggest a major community reorganization, potentially driven by rising seawater temperatures and other environmental changes. This study highlights the importance of long-term monitoring using multiple indicators to detect and interpret climate-driven biodiversity shifts in coastal marine ecosystems.

## Introduction

Climate change has extensively impacted marine ecosystems, influencing ecosystem functions through shifts in biodiversity (Doney et al., 2012; Poloczanska et al., 2013). Numerous previous studies have highlighted the serious effects of global warming and climate change on biological communities (Pinsky et al., 2013; Hongo & Yamano, 2013). Rising sea surface temperatures, in particular, have altered species distributions and diversity, with warm-adapted species expanding their ranges and cold-adapted species contracting or shifting their ranges (Pinsky et al., 2013). In Sekisei Lagoon, Okinawa, anomalously high sea surface temperatures during the summer of 2016 caused severe coral bleaching. More than 98% of colonies belonging to 10 of 11 dominant species bleached, and even massive *Porites* exhibited ∼58% bleaching, leading to an extensive decline in coral cover (Nakamura, 2017; Hughes et al., 2017). Beaugrand et al. (2002) has also documented long-term decline and distributional shifts in plankton communities of the North Atlantic, including northward expansions of warm-water taxa and contractions of cold-water taxa. Because sea slugs respond rapidly to temperature fluctuations and environmental change, they provide an effective model for detecting climate-driven shifts in marine ecosystems. Therefore, this study surveyed sea slugs.

The clade Heterobranchia is recognized as one of the most diverse groups in the Gastropoda. They occur across a broad range of marine environments, from tropical to polar regions, and exhibit remarkable morphological and ecological diversity (Jörger et al., 2010). Their feeding on sponges, cnidarians, bryozoans, tunicates, and algae helps to regulate prey populations and maintains benthic community structure (Nimbs & Smith, 2018; Kaligis et al., 2018). Marine, shell-less, or shell-reduced representatives, commonly known as sea slugs, are the focus of this study. Sea slugs respond rapidly to environmental changes because of their short life cycles, narrow habitat preferences, and strong dependence on seawater temperature. These characteristics make them reliable biological indicators of climate-driven shifts in marine ecosystems (Nimbs & Smith, 2018).

Although global research on sea slugs has increased in recent decades, systematic and ecological studies remain far less numerous than those of fishes, corals, or other benthic invertebrates. This pattern is especially pronounced in Japan, where most sea slug studies have been conducted only once and rely heavily on visual records by recreational divers (Goddard, Gosliner & Pearse, 2011; Kashio et al., 2021; Ota et al., 2021; Andrimida, 2022; Pontes et al., 2025). Regional faunal surveys, such as those from the southern coast of Miyazaki Prefecture (Taguchi & Yoshida, 2024) and the adjacent waters of the Kujukushima Islands in Nagasaki (Morozumi et al., 2021), further demonstrate that most Japanese studies are single, site-specific inventories, rather than systematic long-term monitoring programs. The northwestern coast of Kyushu, Japan, faces diverse marine influences, including the East China Sea, the Genkai Sea, and the Tsushima Strait. Interactions among these elements foster rich biodiversity and complex ecosystems (Fujikura et al., 2010; Itsukushima & Kano, 2022). In this coastal region, two previous studies have investigated sea slug community structures, one by Matsubayashi (1989) conducted during the 1960s–1980s, and another by Kawahara (2003) in 2003. In the present study, we focused on Tatsunokuchi and Akase in Nomozaki, located in northwestern Kyushu, Japan, near sites used in these earlier surveys. We conducted extensive visual surveys by SCUBA diving. By comparing species occurrence data obtained from the literature and our present study, we documented long-term changes in sea slugs species composition and biodiversity.

### Materials & Methods

Portions of this text were previously published as part of a preprint (https://doi.org/10.1101/2025.07.31.668013).

### Study sites

**Figure 1:**
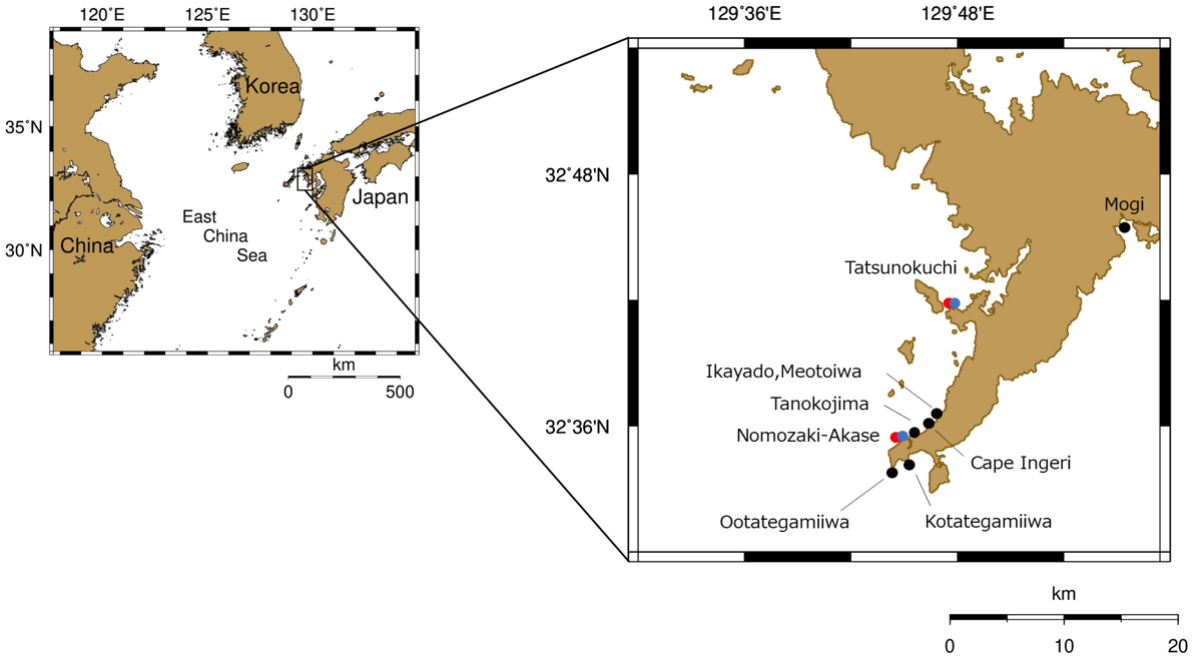
Survey sites of heterobranch sea slug assemblages. Black dots indicate sites surveyed by Matsubayashi (1989). Blue dots indicate sites surveyed by Kawahara (2003), and red dots indicate sites surveyed in the present study. Photographs of Kawahara (2003) and the present study sites (Tatsunokuchi and Nomozaki-Akase) are shown in Fig. S1.

Surveys were conducted at two coastal sites in northwestern Kyushu, Japan, Tatsunokuchi and Nomozaki-Akase in Nagasaki Prefecture (Fig. 1). These sites were selected because they correspond to locations surveyed in previous studies, allowing temporal comparisons of sea slug assemblages. Both sites face the East China Sea and are influenced by the warm Tsushima Current, representing a warm-temperate coastal environment. The coastlines are characterized by rocky reefs interspersed with small patches of sandy substrate and support well-developed macroalgal communities. Water depth in the surveyed areas ranges from approximately 3 to 15 m.

### Historical faunal records

The survey conducted by Matsubayashi (1989) is thought to have been conducted from the 1960s to the 1980s, although the specific timing and frequency of sampling was not documented. The study covered a wide geographic area, including the offshore islands in Nagasaki Prefecture, Japan. To compare sea slugs across the decades, data from sites geographically close to the present study (Mogi, Nomozaki, Kodatejin-iwa, Odatejin-iwa, Tanoko-jima, Takahama Ingeri-bana, and Meoto-iwa) were extracted (Table S1). Sea slugs were collected manually from various microhabitats, including surfaces and undersides of rocks, crevices, and tide pools, as well as from macroalgal beds. Additional methods included sieving sand brought back from octopus pot fishing and collecting by-catch mollusks during shrimp-trawl sorting operations. Because the original taxonomic framework used by Matsubayashi (1989) was not documented, all species names reported in the study were re-evaluated and standardized according to the current WoRMS/MolluscaBase taxonomy (accessed 2025). Species identification in the original study was based on morphological characters, including radula structure.

The survey conducted by Kawahara (2003) was compiled as a master’s thesis at the Faculty of Fisheries, Nagasaki University, but is not publicly available; thus, it is referred to here as unpublished. That survey was conducted from April 2001 to September 2003 using a combination of free diving, SCUBA diving, and intertidal collection. Although sampling was conducted at six sites in northwestern Kyushu, Japan, for temporal comparison in this study, we extracted data only from two of them (Tatsunokuchi and Nomozaki-Akase), due to the completeness of the dataset and their geographic correspondence with the present survey (Fig. 1 and Table S1). Kawahara’s identifications were originally based on the morphological classification. To ensure consistency across survey periods, all historical species names were standardized to current Heterobranchia taxonomy using WoRMS. According to the original notes, the 2001–2003 surveys were also conducted through non-systematic (random) SCUBA dives and free searching, making them methodologically comparable to our present survey. A complete species list is provided in Table S1.

### SCUBA diving survey

All surveys conducted in 2023–2024 were based on random SCUBA dives, during which divers performed comprehensive visual searches for sea slugs. At each site, one or two dives were conducted, each lasting 40–80 min. A free-search method was employed, targeting various microhabitats such as rocky reefs, boulders, and macroalgal beds. In total, 27 SCUBA dives were conducted during the 2023–2024 surveys, corresponding to 25.45 h of underwater observation. Two divers participated in each survey. One diver was responsible for photographing sea slugs underwater using an OLYMPUS Tough TG-6 digital camera, while the other located individuals. Species identification was conducted by a single researcher. When individuals were too small to identify reliably in *situ* or when underwater conditions prevented good photography, a limited number of specimens were collected. These specimens were later examined under a stereomicroscope in the laboratory to confirm species identity. Species identification was conducted based on morphological characteristics, referring to identification guides by Nakano (2019) and Gosliner, Behrens & Valdés (2008). When species-level identification was not possible, specimens were recorded as Genus sp., and when morphological similarity suggested a likely species, but confirmation was uncertain, they were indicated as Genus cf. species. Taxonomic names and higher-level classification followed the WoRMS/MolluscaBase framework, based on Bouchet et al. (2017). In addition to biological data, environmental parameters such as water temperature, visibility, and substrate type were recorded immediately after each dive (Table S2). Surveys were conducted for academic research purposes with official permission granted by the Nagasaki Prefectural Fisheries Division (Permit Nos. 5Gyoshin-Kyo-115 and -116). All dives were conducted under the supervision of a certified diving instructor for safety management.

### Data analysis

Species diversity was compared using data collected by Kawahara (2003) from 2001 to 2003 and our survey data from 2023 to 2024. Data from Matsubayashi (1989) were excluded from diversity index calculations and comparative analyses due to the lack of individual counts. To evaluate species diversity, we calculated, the Shannon–Wiener diversity index (H) and Simpson’s diversity index (D) following definitions in Mittelbach & McGill (2023). Relative abundance was derived from the proportion of individuals of each species in each sample. We calculated both indices because they capture complementary aspects of community structure. Shannon’s index is more sensitive to species evenness and overall diversity, whereas Simpson’s index emphasizes dominance and is less influenced by rare species. Using both indices therefore provides a more comprehensive characterization of temporal shifts in sea slug community structure.

To evaluate temporal changes in species composition, we calculated the relative abundance of each species during each survey period (2001–2003 and 2023–2024), defined as the proportion of individuals of each species compared to the total number of individuals. Fisher’s exact tests were conducted on species-specific count data to assess differences in relative proportions between the two periods. Resulting *p*-values were adjusted using the Benjamini–Hochberg procedure to control the false discovery rate (FDR).

To compare temporal changes in climatic preferences of sea slug species, all recorded species were categorized into four climate affinity groups based on their primary distributional ranges: (1) tropical–subtropical, (2) temperate, (3) tropical–subtropical–temperate, and (4) subarctic–arctic. This classification scheme was adapted from the framework proposed by Grandéz, Ampuero & Barahona (2023) for nudibranchs, with modifications to better reflect distributional characteristics of species in Japanese coastal waters. In particular, species primarily distributed in northern Hokkaido and the Russian Far East were categorized as “subarctic–arctic”. Species names and distributions were verified against WoRMS (WoRMS Editorial Board, 2025; World Register of Marine Species) and relevant primary literature, and supplemented with regional records from Ono & Kato (2020). Species distributed mainly in the Indo-West Pacific, central Pacific, or tropical Pacific were classified as tropical–subtropical; those mainly found in the temperate zones of Japan, the Korean Peninsula, and Hong Kong were categorized as temperate. Species with wide distributions spanning multiple climatic zones, *e.g.*, Indo-Pacific, Indo-West Pacific to temperate Japan, were assigned to the tropical–subtropical–temperate group. The same classification criteria were applied to species recorded in past surveys (Matsubayashi, 1989; Kawahara (2003)). Species for which primary distribution ranges could not be verified due to lack of records in modern literature or databases were excluded from the climatic group analysis. A full list of species with their assigned climatic preferences is provided in Tables S1 and S3.

To evaluate the similarity of species composition among the three survey periods (1960–1980, 2001–2003, and 2023–2024), we calculated the Jaccard similarity coefficient, a commonly used index of community similarity (Legendre & Legendre, 2012). Lower values indicate little overlap in species composition, whereas higher values indicate greater similarity. This index was used to compare temporal shifts in sea slug assemblages.

## Results

**Figure 2:**
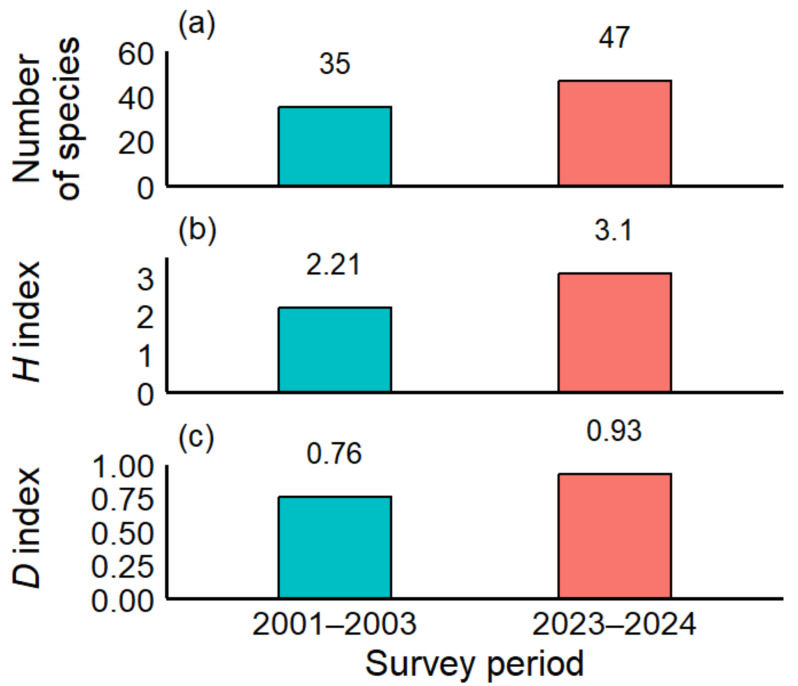
Comparison of heterobranch sea slug assemblages in the past (2001–2003) and the present (2023–2024). (A) Numbers of species, (B) Shannon-Wiener diversity index (*H*), and (C) Simpson diversity index (*D*). Both surveys were conducted at Tatsunokuchi and Nomozaki-Akase on the west coast of Kyushu, Japan.

In our SCUBA surveys conducted during 2023 and 2024, a total of 27 survey dives conducted by two divers (equivalent to 25.45 h) yielded 47 species and 304 individuals (Tatsunokuchi: 30 species, 169 individuals; Nomozaki-Akase: 32 species, 135 individuals). A total of 24 species were newly recorded in the 2023–2024 surveys, and 17 of these (71%) were tropical to subtropical species (Table 1). In contrast, the 2001–2003 survey recorded 35 species (Tatsunokuchi: 25 species, 313 individuals; Nomozaki-Akase: 21 species, 189 individuals) (Fig. 2A). The Shannon–Wiener diversity index (H) increased from 2.21 in 2001–2003 to 3.10 in 2023–2024, and the Simpson diversity index (D) increased from 0.76 to 0.93 during the same period (Figs. 2B, 2C). These higher index values indicate greater species diversity in recent surveys; however, because diversity indices are non-linear, these values should be interpreted descriptively rather than as proportional increases.

**Table 1:** List of 24 heterobranch sea slug species newly recorded in coastal waters of northwestern Kyushu during the 2023–2024 survey. Species not documented in previous literature (Matsubayashi, 1989; Kawahara, 2003) are considered newly recorded for the region. Unidentified morphospecies were labeled as “sp. 1”, “sp. 2”, *etc*., following the numbering system used in Ono & Kato (2020).

**Scientific name**	**Location**	**Data observed**	**Climate classification**
*Stiliger* sp.	Tatsunokuchi	Jul and Oct 2023	Tropical–subtropical
*Thuridilla splendens* (Baba,1949)	Tatsunokuchi ^.^ Nomozaki-Akase	Oct 2023 and Jan 2024	Tropical–subtropical
*Elysia asbecki* Wägele, Stemmer, Burghardt & Händeler, 2010	Nomozaki-Akase	Jan 2024	Tropical–subtropical
*Elysia lobata* A. Gould, 1852	Nomozaki-Akase	Jan 2024	Tropical–subtropical
*Elysia japonica* Eliot, 1913	Tatsunokuchi ^.^ Nomozaki-Akase	Jan 2024	Temperate species
*Thuridilla vataae* (Risbec, 1928)	Nomozaki-Akase	Jan 2024	Tropical–subtropical
*Thuridilla albopustulosa* Gosliner, 1995	Nomozaki-Akase	Jul and Oct 2023	Tropical–subtropical
*Chelidonura hirundinina* (Quoy & Gaimard, 1833)	Tatsunokuchi	Jun 2023	Tropical–subtropical
*Bermudella japonica* (Baba, 1949)	Tatsunokuchi	Jan 2024	Temperate species
*Diaphorodoris mitsuii* (Baba, 1938)	Tatsunokuchi	Jan 2024	Tropical–subtropical
*Cadlinella ornatissima* (Risbec, 1928)	Tatsunokuchi	Aug and Sep 2023	Tropical–subtropical
*Goniobranchus geometricus* (Risbec, 1928)	Tatsunokuchi	Oct 2023	Tropical–subtropical
*Goniobranchus aureopurpureus* (Collingwood, 1881)	Nomozaki-Akase	Aug 2023	Tropical–subtropical
*Mexichromis multituberculata* (Baba, 1953)	Tatsunokuchi	Aug 2023	Tropical–subtropical
*Hypselodoris placida* (Baba, 1949)	Nomozaki-Akase	Jul 2023	Temperate species
*Phidiana anulifera* (Baba, 1949)	Nomozaki-Akase	Nov 2023	Tropical–subtropical
*Caloria indica* (Bergh, 1896)	Tatsunokuchi	Jan 2024	Tropical–subtropical– temperate species
*Bulbaeolidia alba* (Risbec, 1928)	Tatsunokuchi	Jan 2024	Tropical–subtropical
*Phyllodesmium magnum* Rudman, 1991	Nomozaki-Akase	Sep 2023	Tropical–subtropical– temperate species
*Scyllaea pelagica* Linnaeus, 1758	Nomozaki-Akase	Oct 2023	Tropical–subtropical
*Polycera* sp. 7	Nomozaki-Akase	Jan 2024	Temperate species
*Marionia* sp. 1	Tatsunokuchi	Jan 2024	Temperate species
*Tenellia* sp. 44	Tatsunokuchi	Jan 2024	Tropical–subtropical
Flabellina sp. 3	Tatsunokuchi	Aug 2023	Tropical–subtropical

**Figure 3:**
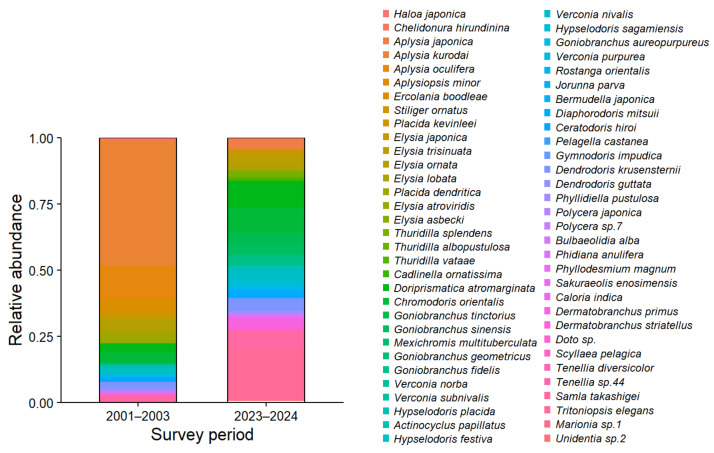
Comparative heatmap of the relative abundance of heterobranch sea slug assemblages in the past (2001–2003) and present (2023–2024). Relative abundance was calculated as the proportion of individuals of each species to the total number of individuals observed during each period. Both surveys were conducted at Tatsunokuchi and Nomozaki-Akase on the west coast of Kyushu, Japan.

**Figure 4:**
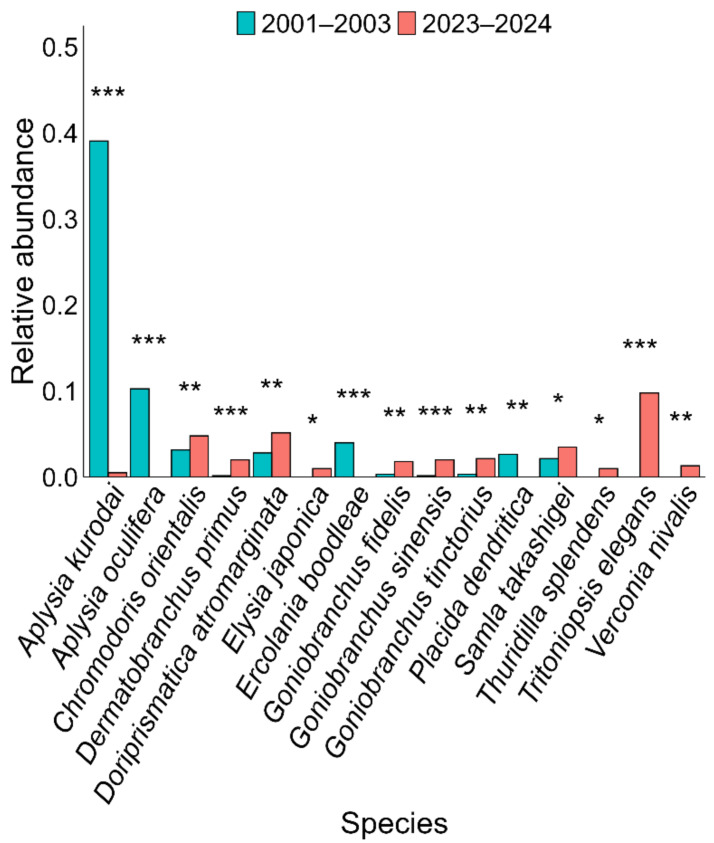
Comparison of relative abundances of 15 heterobranch sea slug species that showed statistically significant differences between the past (2001–2003) and present (2023–2024). Asterisks indicate significance levels: *p < 0.05, **p < 0.01, ***p < 0.001. Both surveys were conducted at Tatsunokuchi and Nomozaki-Akase on the northwestern coast of Kyushu, Japan.

Community structure, based on the relative abundances of individual species, showed a clear shift between 2001–2003 and 2023–2024 (Fig. 3). In the earlier period (2001–2003), a few dominant species, such as *Aplysia kurodai* and *Aplysia oculifera*, accounted for a large proportion of the community, with *Aplysia kurodai* alone comprising ∼45% of all individuals. In contrast, in 2023–2024, the relative abundance of these dominant species had declined markedly. Instead, species such as *Tritoniopsis elegans, Doriprismatica atromarginata, Goniobranchus orientalis, Goniobranchus sinensis, and Goniobranchus tinctorius* had increased in proportion, indicating a shift toward a more diverse community. These compositional shifts were further supported by statistical analyses, which identified 15 species exhibiting significant changes in relative abundance between the 2001–2003 and 2023–2024 periods (Fig. 4).

**Figure 5:**
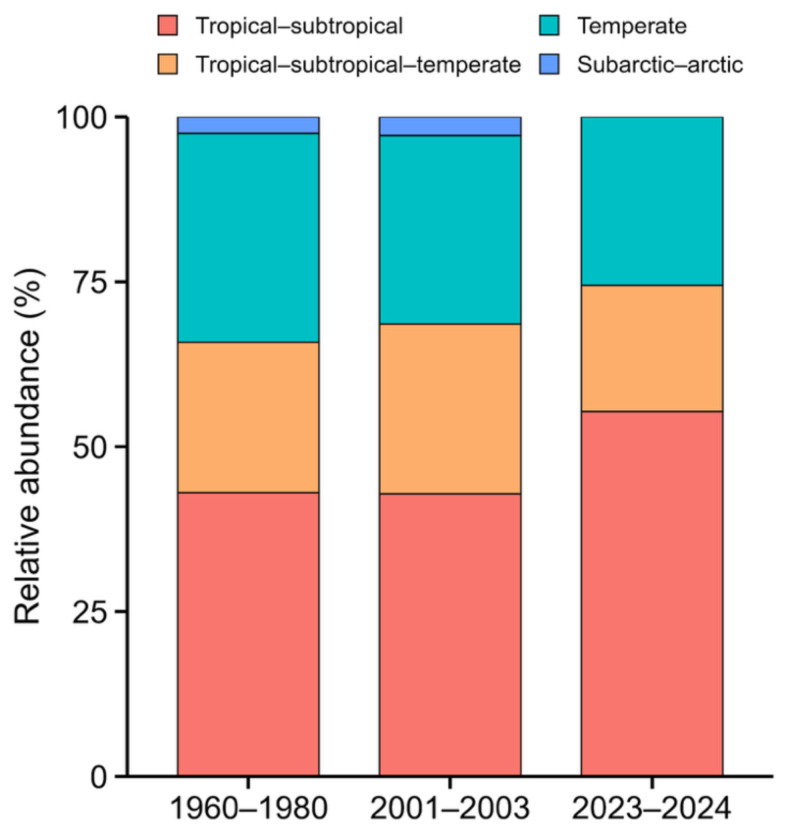
Comparison of climatic preferences of observed heterobranch sea slug species during different survey periods. In the most recent period, species belonging to the subarctic–arctic group were no longer detected, while the proportion of tropical–subtropical species increased. All surveys were conducted along the northwestern coast of Kyushu, Japan.

Comparison of species composition by climatic preferences revealed that among the 47 species observed in the present study (2023–2024), 55.3% (26 species) were tropical–subtropical, 19.2% (9 species) were tropical–subtropical–temperate, and 25.5% (12 species) were temperate. In contrast, among the 79 species recorded between 1960 and 1980 (Matsubayashi, 1989), 43.0% (34 species) were tropical–subtropical, 22.8% (18 species) were tropical–subtropical–temperate, 31.6% (25 species) were temperate, and 2.5% (2 species) were subarctic–arctic. Similarly, among the 35 species recorded approximately 20 years ago (Kawahara (2003)), 42.9% (15 species) were tropical–subtropical, 25.7% (9 species) were tropical–subtropical–temperate, 28.6% (10 species) were temperate, and 2.9% (1 species) were subarctic–arctic. These results indicate increasing proportions of tropical–subtropical species and decreasing proportions of temperate species (Fig. 5).

The number of sea slug species observed in each time period was 47 species in 2023–2024, 35 species in 2001–2003, and 79 species in 1960–1980. Among the three periods, the lowest similarity in species composition was found between 2023–2024 and 1960–1980 (Jaccard Coefficient (CC): 0.105) (Table 2). In contrast, the similarity between 2001–2003 and 1960–1980 was relatively higher (CC: 0.281) (Table 2).

## Discussion

The sea and its sea slugs have undergone dramatic changes during the past 50 years, almost appearing as entirely different worlds. In this study, we quantitatively and qualitatively compared sea slug communities across three time periods (1960–1980, 2001–2003, and 2023–2024) to reveal long-term shifts in community structure. Compared to 20 years ago, diversity indices (Shannon–Wiener diversity index (*H*) and Simpson diversity index (*D*)) have increased (Fig. 2), and statistically significant changes in species composition ratios were observed in 15 species, indicating dynamic alterations at the species level. During the 2023–2024 survey, several species that had not been recorded in the 2001–2003 survey were detected. Notably, approximately 70% of these newly recorded species were tropical–subtropical taxa, contrasting with the historical species composition documented two decades earlier and indicating a clear shift in community structure likely driven by climate warming (Table 1). Heatmap analyses (Fig. 3) revealed clear shifts in community structure, with distinct differences in species composition between time periods. Similarity analyses using the Jaccard Coefficient of Commonality (CC) showed the greatest community dissimilarity between the 1960–1980 and 2023–2024 periods (Table 2), suggesting a major community turnover. Similar long-term observations using sea slugs have also been conducted in Australia (Nimbs & Smith, 2018; Nimbs et al., 2020), but in temperate coastal waters of Japan, this study provides one of the first long-term assessments of marine community change using sea slugs as bioindicators.

**Figure 6:**
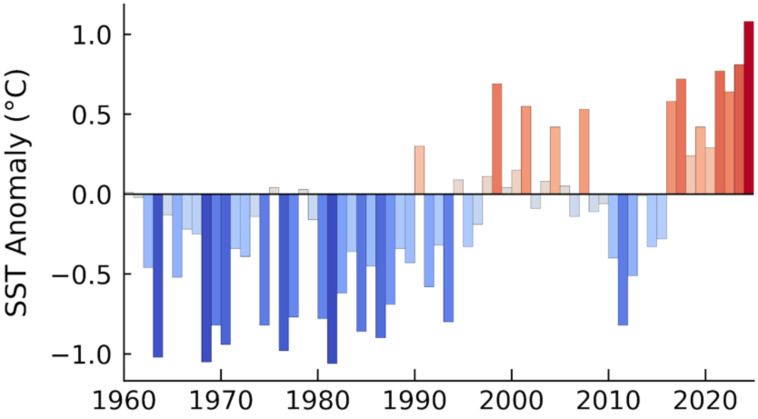
Annual sea surface temperature (SST) anomalies in the study region from 1960 to 2024. Each bar represents the deviation (in °C) from the 1991–2020 climatological average. Red bars indicate warmer-than-average years, while blue bars indicate cooler-than-average years. The height of each bar corresponds to the magnitude of the anomaly.

Why has the composition of sea slug assemblages changed so dramatically? The most plausible explanation lies in recent climate change, particularly the rise in seawater temperature. In fact, long-term sea surface temperature (SST) anomalies in the study area have shown a marked and persistent warming trend, especially since the late 2010s (Fig. 6). Since the 1980s, SSTs have steadily increased, with strong positive anomalies evident in recent years. Such thermal changes drive poleward range expansions of tropical and subtropical species (Poloczanska et al., 2013), and this pattern is clearly reflected in our data. Of the 47 sea slug species recorded in 2023–2024, 55.3% were classified as tropical–subtropical species, substantially higher than proportions observed in 1960-1980 (43.0%) and 2001–2003 (40.0%). Shallow-water sea slugs are particularly sensitive to water temperature and exhibit rapid shifts in occurrence patterns in response to thermal variability (Sanford et al., 2019; Poloczanska et al., 2013). In addition to temperature, other environmental factors likely contribute to community restructuring, including fluctuations in food availability, *e.g.*, algae, hydroids, and encrusting organisms, changes in benthic substrate composition, and anthropogenic impacts such as coastal development (Chen et al., 2022). While rising seawater temperatures driven by climate change are the primary factor, shifts in seaweed communities, which serve as critical food resources, cannot be ignored. Along the coast of western Japan, extensive declines in perennial canopy-forming brown algae and expansion of subtropical species have been documented (Haraguchi et al., 2009; Tanaka et al., 2012), resulting in seasonal algal beds that diminish markedly in autumn. Such changes likely reduce food availability for herbivorous mollusks, such as the sea hare *Aplysia kurodai*, and may be associated with localized population declines. These factors act synergistically, suggesting that the recent reorganization of sea slug assemblages is the result of a complex interplay between abiotic environmental changes and biotic interactions.

An important observation in this study is the emergence of several sea slug species never recorded in this region during any previous surveys. The high proportion of tropical and subtropical species among newly recorded taxa suggests that warm-water populations transported from offshore areas during summer may increasingly colonize coastal habitats in northwestern Kyushu, Japan (Sanford et al., 2019). Similar poleward expansions and community impacts of warm-affinity species have also been documented in other temperate regions (Sorte, Williams & Carlton, 2010). Such poleward range expansions and climate-driven shifts in biodiversity have been reported globally (Poloczanska et al., 2013; Burrows et al., 2011), and the present findings suggest that similar community-level changes are already underway in this region as part of broader warming-driven biogeographic rearrangements (Dornelas et al., 2023; Pinsky, Selden & Kitchel, 2020). The arrival of previously unrecorded species may lead to new competitive and trophic interactions, potentially disrupting existing ecological balances (Sasaki et al., 2024; Zarco-Perello et al., 2020). Newly observed species likely include undescribed or rare taxa, indicating that ecological changes may not be fully captured by conventional taxonomic or ecosystem assessment frameworks (Rogers et al., 2023). This lag in recognition poses challenges for biodiversity monitoring and conservation, emphasizing the urgent need for accurate species identification and systematic data accumulation (Ma et al., 2024; Kerry et al., 2022). While the appearance of new species may superficially suggest increased biodiversity, it often accompanies a reorganization of local communities, including shifts in dominant species or disappearance of previously common taxa, which may trigger ecosystem instability and negative feedback loops (Pecl et al., 2017). These findings underscore the necessity of understanding climate-driven responses not only in terms of tropical species expansion, but also in the context of local extinctions of cold-affinity species, calling for a more holistic view of climate-induced ecological transformation.

**Table 2:** Numbers of shared species and Jaccard Coefficient (CC) for sea slug heterobranch sea slug species observed in northwestern coast of Kyushu, Japan.

**Comparison period**	**Number of shared species**	**Jaccard coefficient**
1960–1980 × 2001–2003	18	0.188
2001–2003 × 2023–2024	18	0.281
1960–1980 × 2023–2024	12	0.105

## Conclusions

Our findings highlight the value of integrating historical datasets with contemporary observations to detect climate-driven biodiversity shifts. This study has certain limitations, particularly in quantitative comparisons with historical data from 50 years ago due to missing individual counts and unspecified survey periods in earlier datasets. Moreover, differences in survey methods and the limited number of sampling sites must also be acknowledged as constraints. The nature of scuba-based surveys introduces additional variability due to diver experience and sea conditions. Indeed, previous studies have reported that observer expertise and experience can influence species detection rates and abundance estimates in underwater visual censuses (Bernard et al., 2013). Sea surface temperature continues to rise due to global warming, and its ecological consequences remain largely unknown. Because historical datasets lacked standardized sampling effort, individual counts, and consistent methods, it remains difficult to quantitatively assess long-term changes in sea slug communities. To address these limitations and improve future predictions of biodiversity shifts, more quantitative and standardized monitoring approaches are essential. Methods such as line transects can reduce observer bias and enhance temporal comparability. Additionally, the use of environmental DNA (eDNA) and image analysis technologies can enable more extensive and objective monitoring of biodiversity. Furthermore, collecting information on the age structure and reproductive status of observed individuals will be valuable in understanding processes of range expansion and local establishment. Continued quantitative, long-term monitoring of coastal ecosystems is vital for developing effective conservation strategies and improving predictions of future ecological shifts.

## Supplemental Information

10.7717/peerj.20870/supp-1 Supplemental Information 1 Survey sites used in this study and in Kawahara (2003) Left: Tatsunokuchi; Right: Nomozaki Akase. The present study area is shown in Fig. 1.

10.7717/peerj.20870/supp-2 Supplemental Information 2 Photograph showing diving surveys conducted in this study

10.7717/peerj.20870/supp-3 Supplemental Information 3 Photographs illustrate the morphological diversity of heterobranch sea slugs recorded from coastal waters of northwestern Kyushu, Japan, during the 2023–2024 underwater surveys

10.7717/peerj.20870/supp-4 Supplemental Information 4 Summary of heterobranch sea slugs data extracted from the literature (A) Matsubayashi (1989); (B) Kawahara (2003). Climatic distribution categories, *e.g.*, tropical-subtropical, warm-temperate, were assigned to each species in this study.

10.7717/peerj.20870/supp-5 Supplemental Information 5 Summary of environmental data recorded during the present scuba diving surveys

10.7717/peerj.20870/supp-6 Supplemental Information 6 Summary of heterobranch sea slug assemblages recorded during the present scuba diving surveys

10.7717/peerj.20870/supp-7 Supplemental Information 7 Heterobranchia raw data

## Additional Information and Declarations

### Field Study Permissions

The following information was supplied relating to field study approvals (i.e., approving body and any reference numbers): Field experiments were approved by Nagasaki University, Nomozaki Sanwa Fisheries Cooperative and the Seihi Southern Fisheries Cooperative for granting us permission to conduct the underwater surveys (Permit Nos. 5Gyoshin-Kyo-115 and -116).

### Data Availability

The following information was supplied regarding data availability: The raw measurements are available in the Supplementary File.
