# Supplementary figures and images for: A different world: temporal changes in the community structure of sea slugs (Heterobranchia) in northwest Japan spanning more than a half-century
Source: PeerJ. 2026 Mar 2;14:e20870. doi: 10.7717/peerj.20870 (PMC12962135; doi:10.7717/peerj.20870)

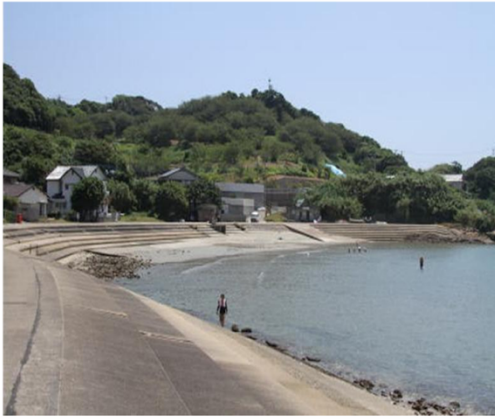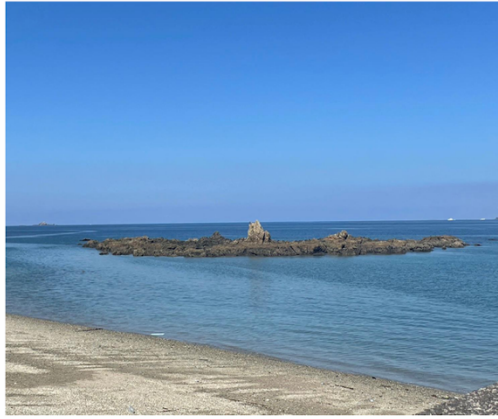

Supplement: Supplemental Information 1 — Left: Tatsunokuchi; Right: Nomozaki Akase. The present study area is shown in Fig. 1. [file peerj-14-20870-s001.pdf]

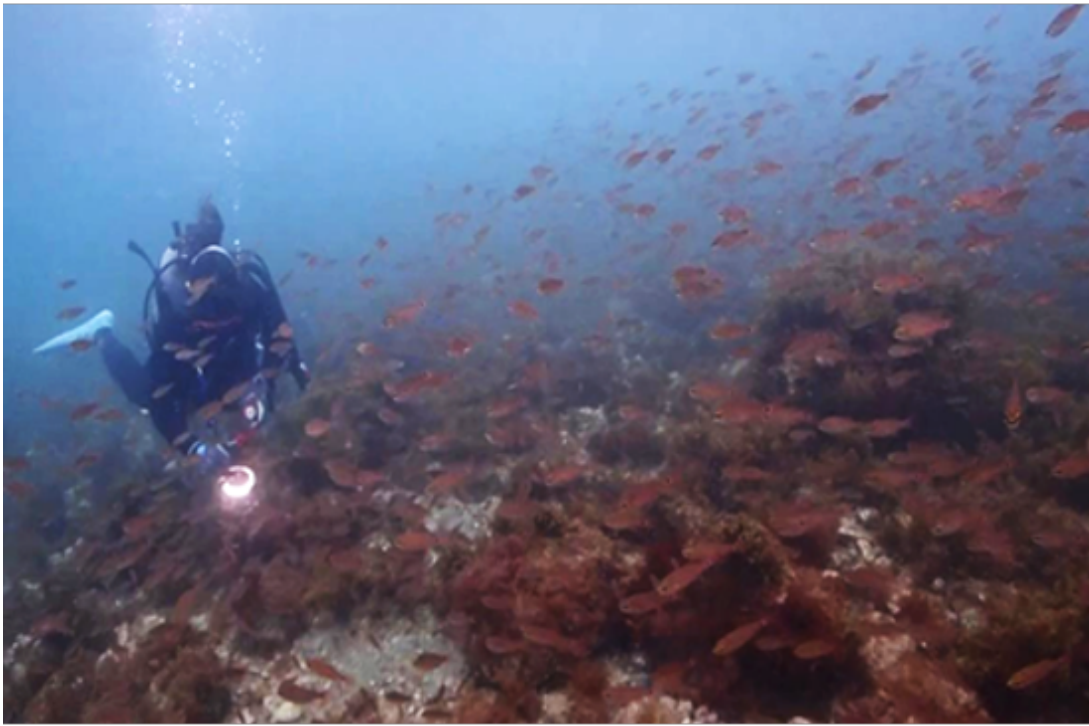

Supplement: Supplemental Information 2 [file peerj-14-20870-s002.pdf]
