# Supplementary material for: A different world: temporal changes in the community structure of sea slugs (Heterobranchia) in northwest Japan spanning more than a half-century
Source: PeerJ. 2026 Mar 2;14:e20870. doi: 10.7717/peerj.20870 (PMC12962135; doi:10.7717/peerj.20870)
Supplement: Supplemental Information 3 [file peerj-14-20870-s003.pdf]

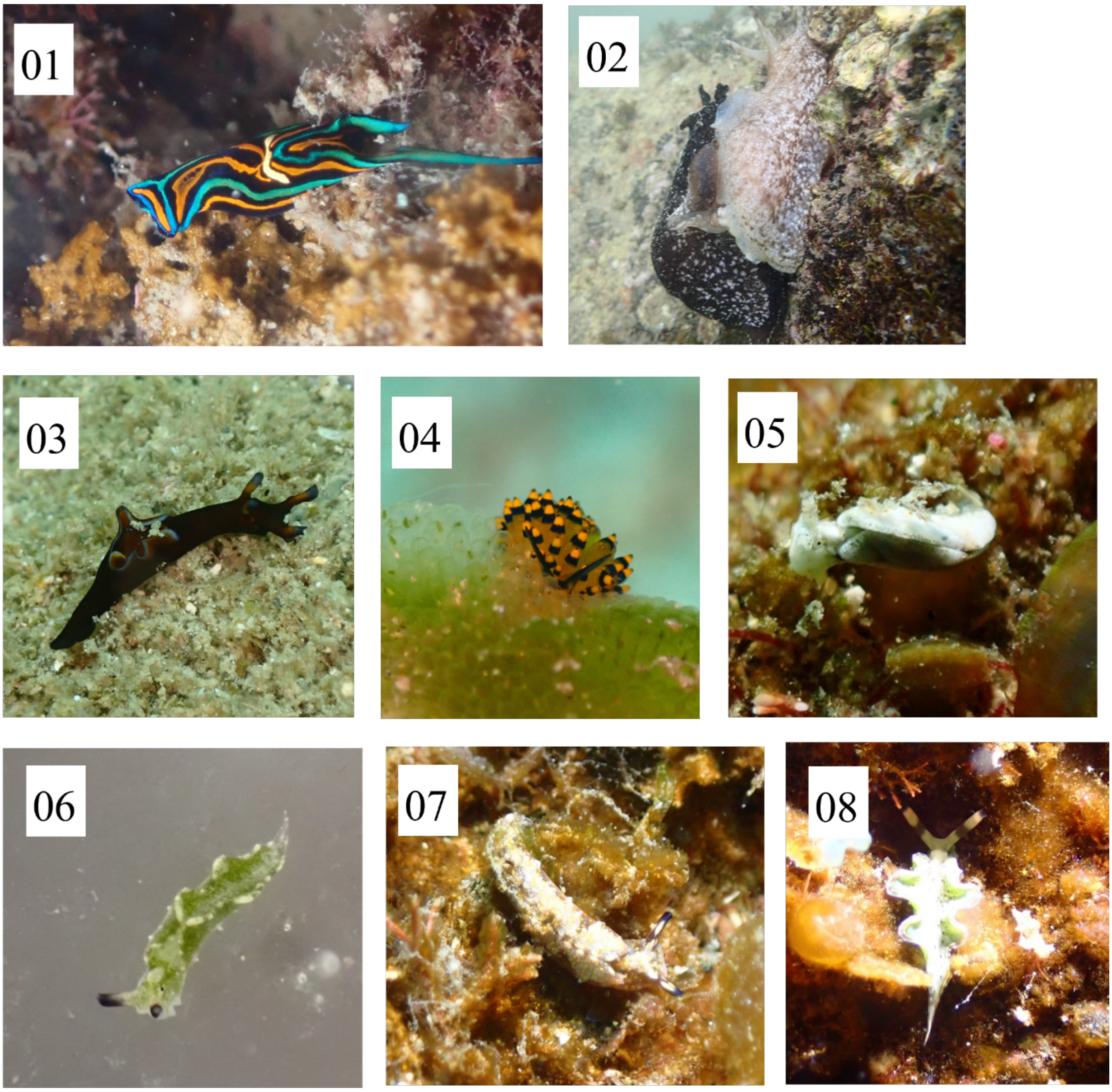


01.*Chelidonura hirundinina* (Quoy&Gaimard, 1833)
02. *Aplysia kurodai* (Baba, 1937)
03. *Aplysia japonica* G. B. Sowerby I, 1869
04. *Stiliger ornatus* Ehrenberg, 1828
05. *Elysia asbecki* Wägele, Stemmer, Burghardt & Händeler, 2010
06. *Elysia atroviridis* Baba,1955
07. *Elysia japonica* Eliot, 1913
08. *Elysia lobata* A. Gould, 1852

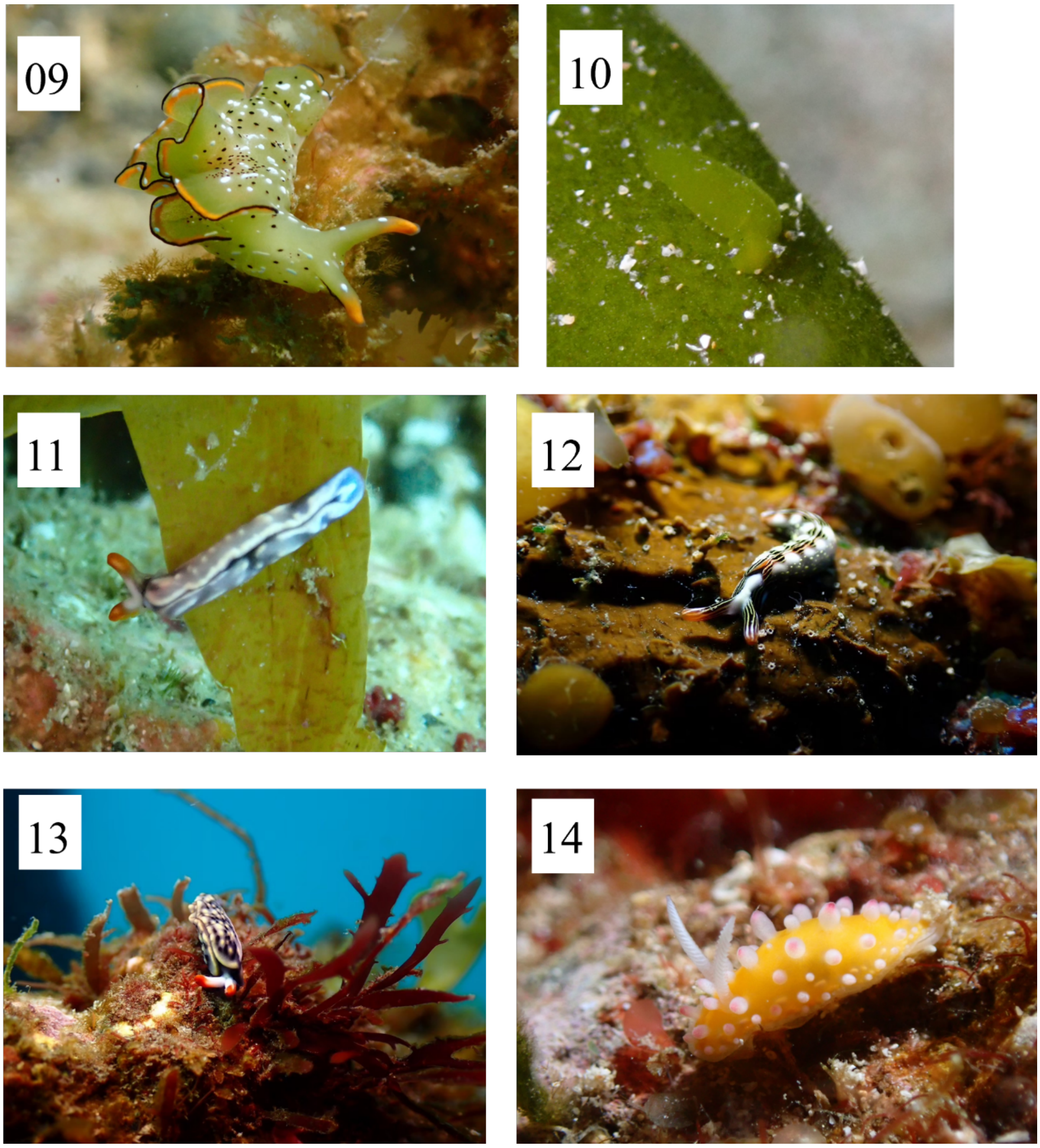


09. *Elysia ornata* (Swainson, 1840)
10. *Elysia trisinuata* Baba, 1949
11. *Thuridilla albopustulosa* Gosliner, 1995
12. *Thuridilla splendens* (Baba,1949)
13. *Thuridilla vataae* (Risbec,1928)
14. *Cadlinella ornatissima* (Risbec,1928)

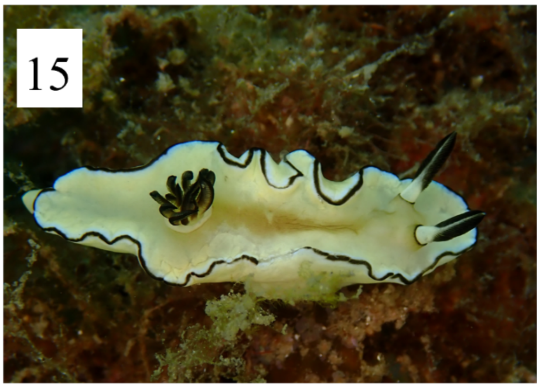


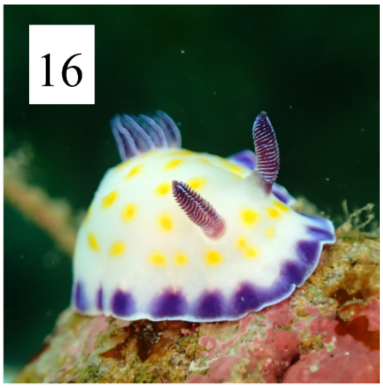


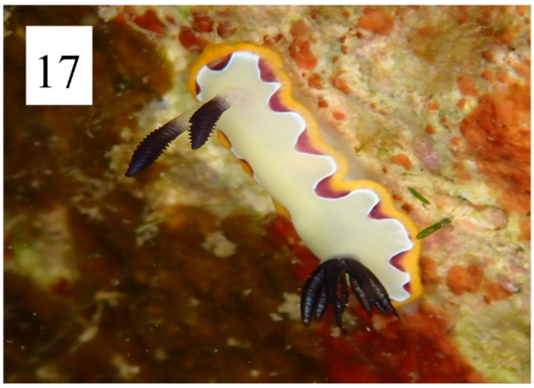


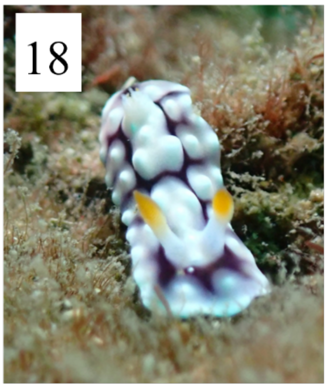


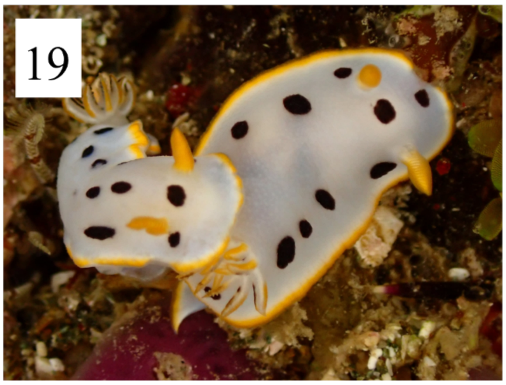


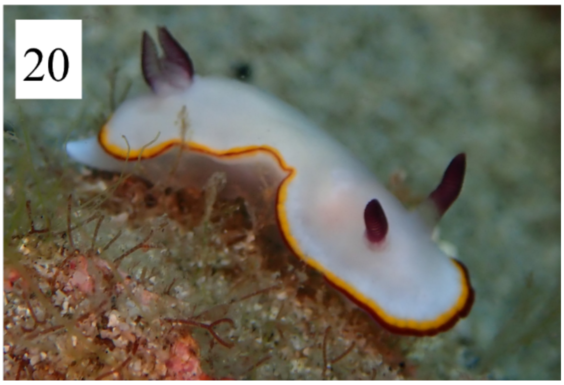


15. *Doriprismatica atromarginata* (Cuvier,1804)
16. *Goniobranchus aureopurpureus* (Collingwood, 1881)
17. *Goniobranchus fidelis* (Kelaart,1858)
18. *Goniobranchus geometricus* (Risbec, 1928)
19. *Chromodoris orientalis* Rudman, 1983
20. *Goniobranchus sinensis* (Rudman,1985)

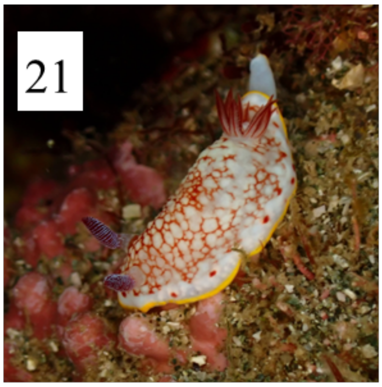

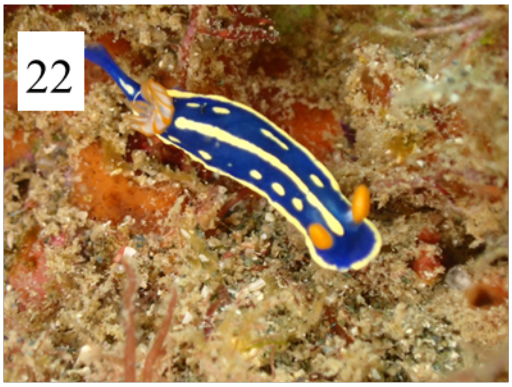

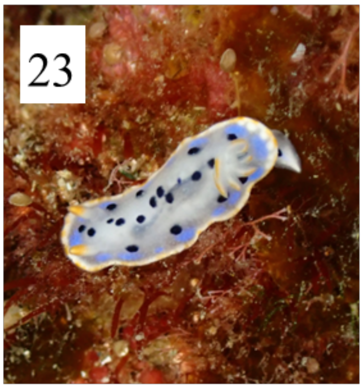

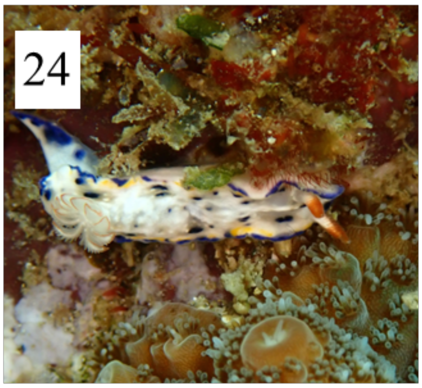

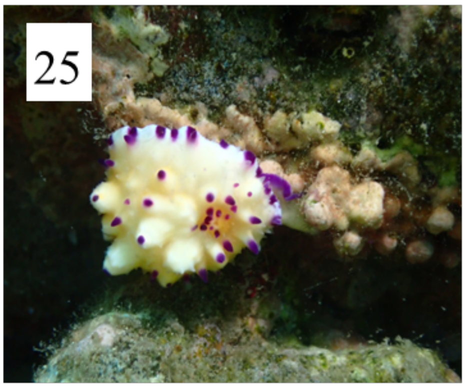

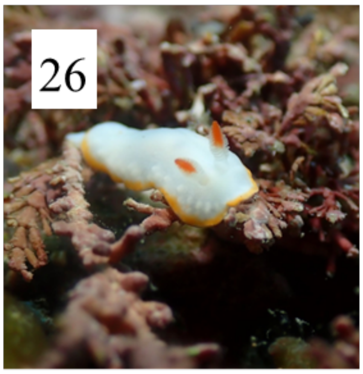

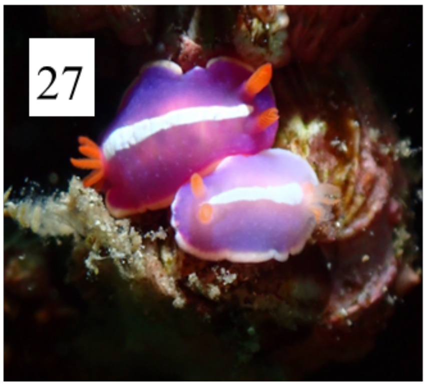

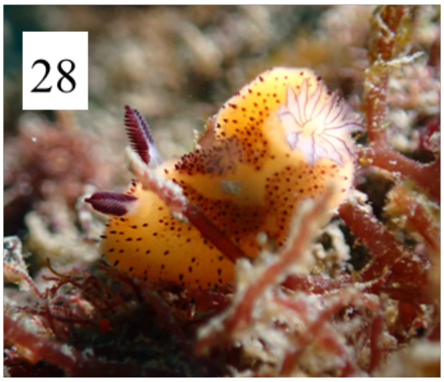

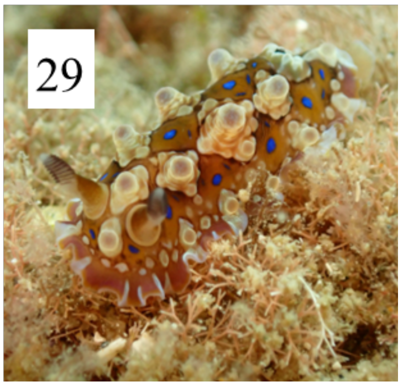


21. *Goniobranchus tinctorius* (Rüppell & Leuckart, 1830)
22. *Hypselodoris festiva* (A.Adams,1861)
23. *Hypselodoris placida* (Baba,1949)
24. *Hypselodoris sagamiensis* (Baba,1949)
25. *Mexichromis multituberculata* (Baba,1953)
26. *Verconia nivalis* (Baba,1937)
27. *Verconia purpurea* (Baba,1949)
29. *Dendrodoris krusensternii* (J. E. Gray, 1850)

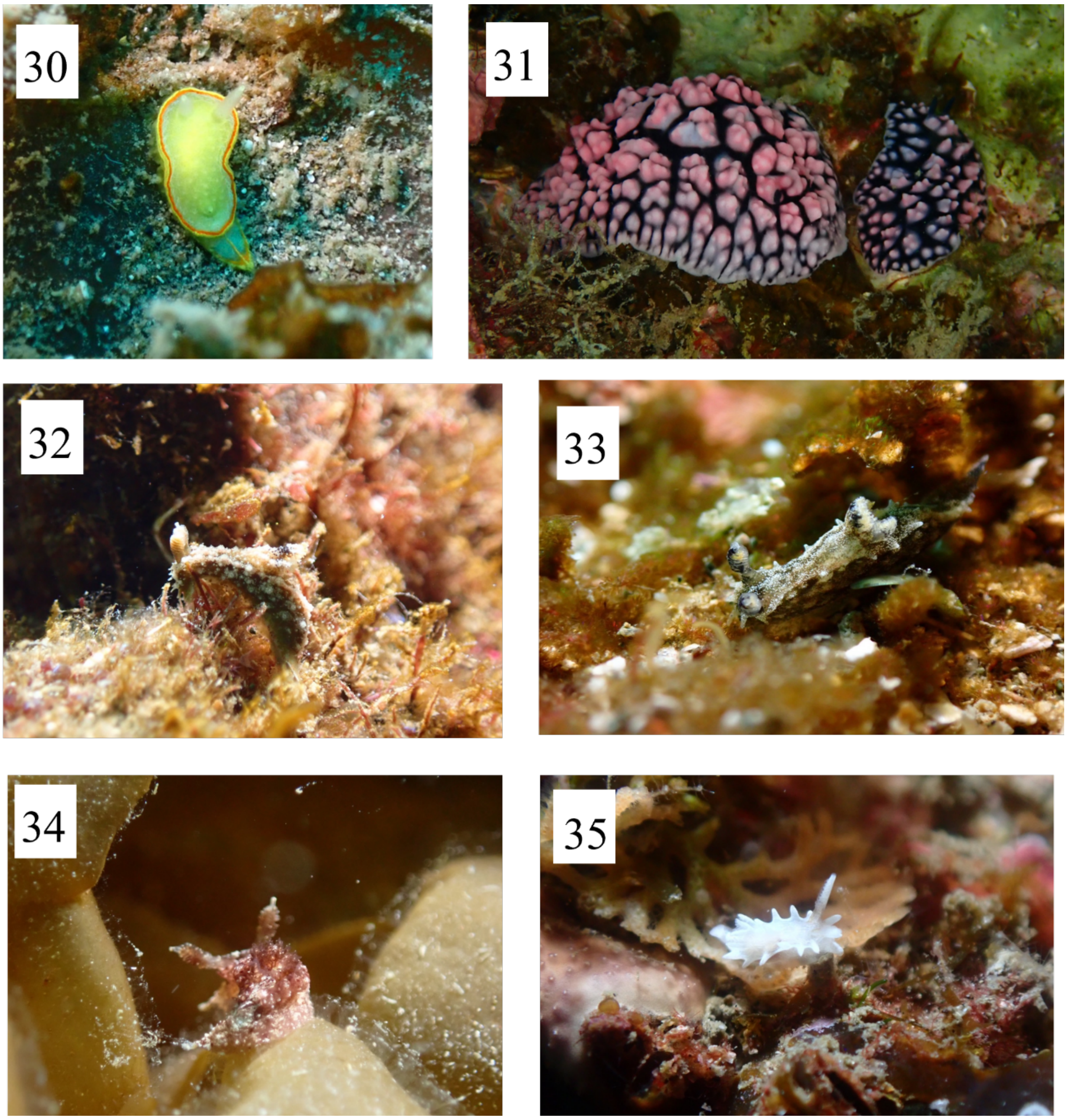


30. *Diaphorodoris mitsuii* (Baba, 1938)
31. *Phyllidiella pustulosa* (Cuvier, 1804)
32. *Polycera japonica* Baba, 1949
33. *Polycera* sp.7
34. *Pelagella castanea* (Alder & Hancock, 1845)
35. *Bermudella japonica* (Baba, 1949)

**Fig. S3** Photographs illustrate the morphological diversity of heterobranchi recorded from coastal waters of northwestern Kyushu, Japan, during the 2023–2024 underwater surveys.

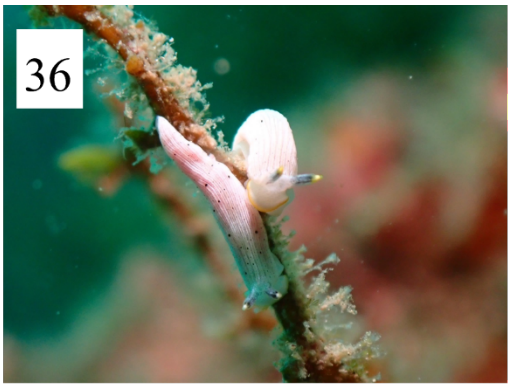

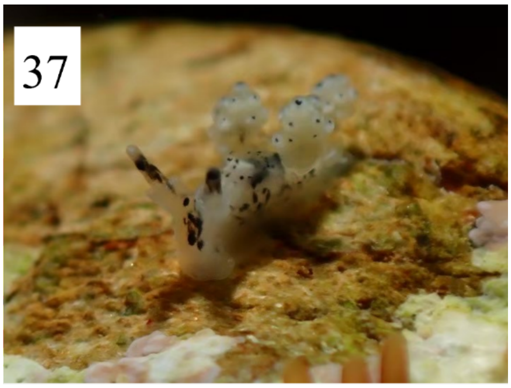

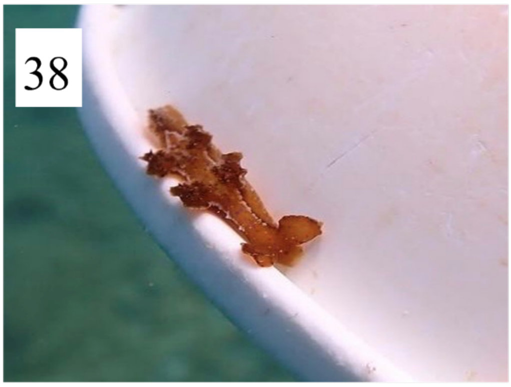

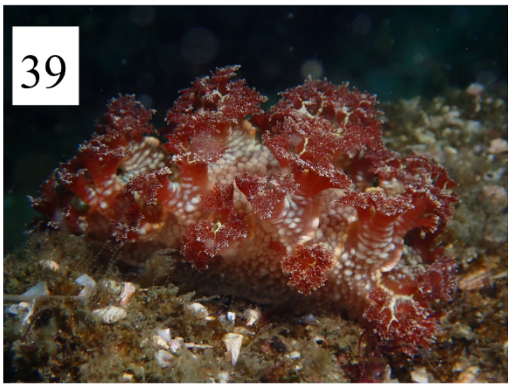

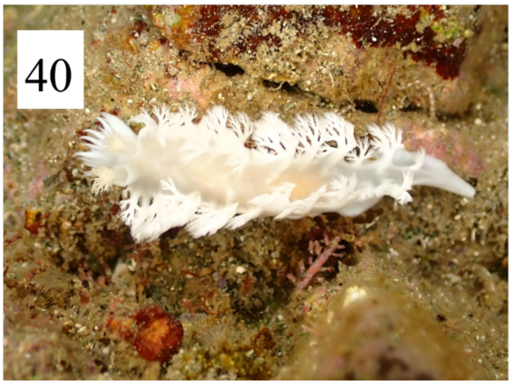

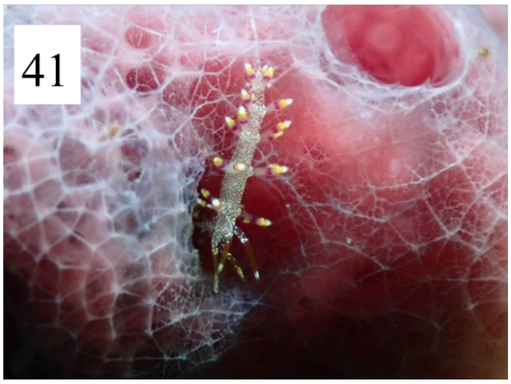


36. *Dermatobranchus primus* (Baba,1976)
37. *Doto sp.*
38. *Scyllaea pelagica* Linnaeus, 1758
39. *Marionia* sp.1
40. *Tritoniopsis elegans* (Audouin, 1826)
41. *Unidentia sp.* 2

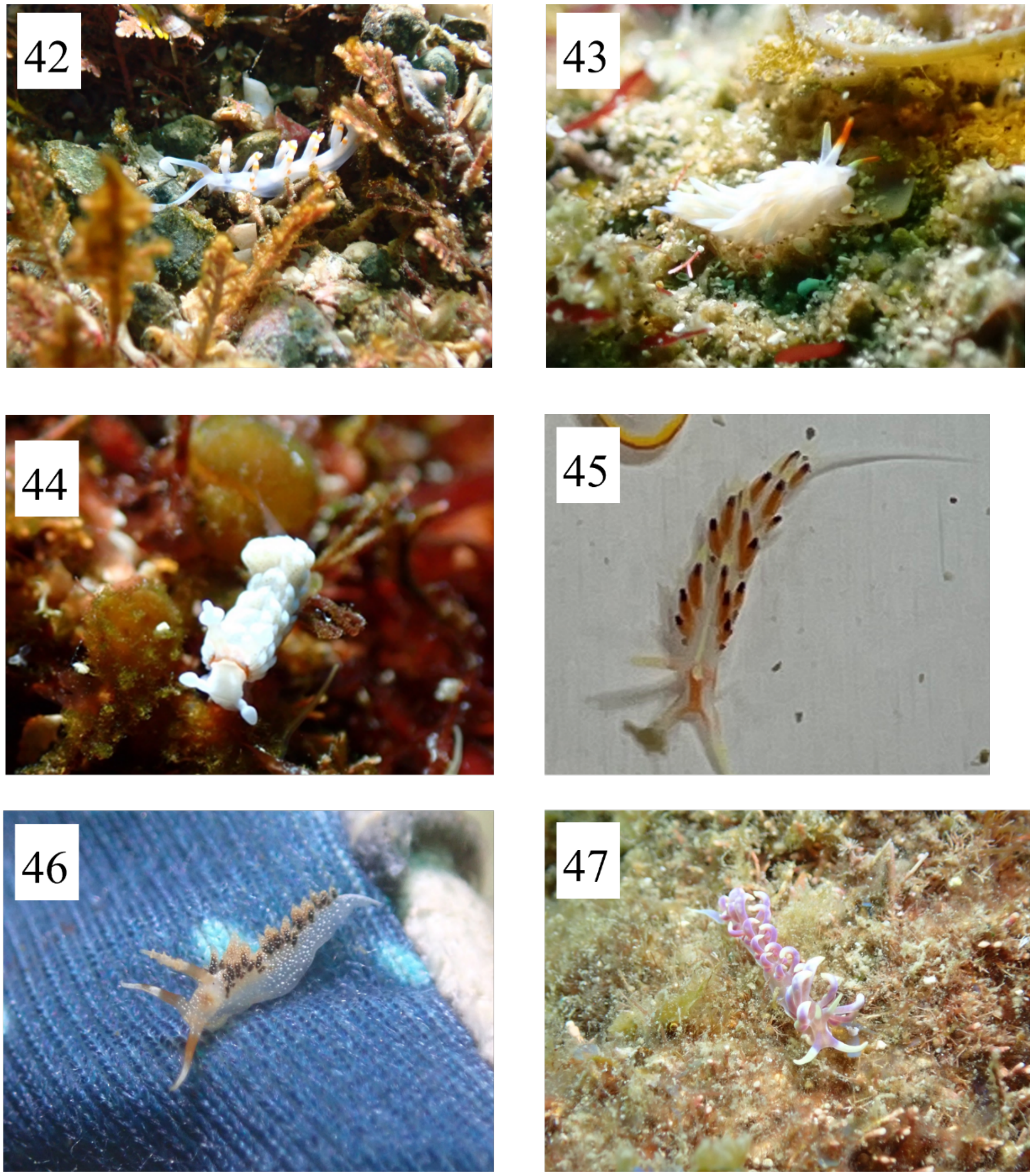


42. Samla takashigei Korshunova, Martynov, Bakken, Evertsen, Fletcher, Mudianta, H. Saito, Lundin, Schrödl & Picton, 2017
43. *Tenellia* sp.44
44. Bulbaeolidia alba (Risbec, 1928)
45. *Caloria indica* (Bergh,1896)
46. *Phidiana anulifera* (Baba, 1949)
47. *Phyllodesmium magnum* Rudman, 1991
