# Supplementary material for: A different world: temporal changes in the community structure of sea slugs (Heterobranchia) in northwest Japan spanning more than a half-century
Source: PeerJ. 2026 Mar 2;14:e20870. doi: 10.7717/peerj.20870 (PMC12962135; doi:10.7717/peerj.20870)
Supplement: Supplemental Information 4 — (A) Matsubayashi (1989); (B) Kawahara (2003). Climatic distribution categories, e.g., tropical-subtropical, warm-temperate, were assigned to each species in this study. [file peerj-14-20870-s004.docx]

1. From Matsubayashi (1989).

| **Site** | **Superorder** | **Order** | **Superfamily** | **Family** | **Species** | **Climate classification** |
| --- | --- | --- | --- | --- | --- | --- |
| Mogi | Nudipleura | Doridida | Polyceroidea | Polyceridae | *Kalinga ornata* Alder & Hancock, 1864 | Tropical–subtropical–temperate species |
|  | Nudipleura | Nudibranchia | Arminoidea | Arminidae | *Armina babai* (S. Tchang, 1934) | Temperate species |
|  | Nudipleura | Pleurobranchida | Pleurobranchoidea | Pleurobranchaeidae | *Pleurobranchaea maculata* (Quoy & Gaimard, 1832) | Tropical–subtropical species |
| Nomozaki |  |  | Acteonoidea | Acteonidae | *Punctacteon fabreanus* (Crosse, 1873) | Tropical–subtropical species |
|  |  | Cephalaspidea | Bulloidea | Tornatinidae | *Acteocina exilis* (Dunker, 1860) | Temperate species |
|  |  | Umbraculida | Umbraculoidea | Umbraculidae | *Umbraculum umbraculum* ([Lightfoot], 1786) | Tropical–subtropical–temperate species |
|  |  | Aplysiida | Aplysioidea | Aplysiidae | *Aplysia japonica* G. B. Sowerby I, 1869 | Temperate species |
|  | Nudipleura | Doridida | Chromodoridoidea | Chromodorididae | *Goniobranchus tumuliferus* (Collingwood, 1881) | Tropical–subtropical species |
|  | Nudipleura | Doridida | Polyceroidea | Polyceridae | *Kaloplocamus acutus* Baba, 1949 | Tropical–subtropical species |
|  | Nudipleura | Doridida | Polyceroidea | Polyceridae | *Crimora lutea* Baba, 1949 | Tropical–subtropical–temperate species |
|  | Nudipleura | Nudibranchia | Arminoidea | Arminidae | *Dermatobranchus ornatus* (Bergh, 1874) | Tropical–subtropical species |
| Kodatagamiiwa |  | Aplysiida | Aplysioidea | Aplysiidae | *Aplysia japonica* G. B. Sowerby I, 1869 | Temperate species |
|  |  | Aplysiida | Aplysioidea | Aplysiidae | *Aplysia kurodai* (Baba, 1938) | Tropical–subtropical species |
|  |  | Aplysiida | Aplysioidea | Aplysiidae | *Dolabella auricularia* ([Lightfoot], 1786) | Tropical–subtropical species |
|  | Sacoglossa |  | Plakobranchoidea | Hermaeidae | *Aplysiopsis nigra* (Baba, 1949) | Temperate species |
|  | Sacoglossa |  | Plakobranchoidea | Limapontiidae | *Placida dendritica* (Alder & Hancock, 1843) | Temperate species |
|  | Sacoglossa |  | Plakobranchoidea | Plakobranchidae | *Elysia atroviridis* Baba, 1955 | Temperate species |
|  | Nudipleura | Doridida | Chromodoridoidea | Chromodorididae | *Chromodoris orientalis* Rudman, 1983 | Tropical–subtropical species |
|  | Nudipleura | Doridida | Chromodoridoidea | Chromodorididae | *Hypselodoris festiva* (A.Adams, 1861) | Temperate species |
|  | Nudipleura | Doridida | Chromodoridoidea | Chromodorididae | *Hypselodoris maritima* (Baba, 1949) | Tropical–subtropical species |
|  | Nudipleura | Doridida | Chromodoridoidea | Actinocyclidae | *Actinocyclus papillatus* (Bergh, 1878) | Tropical–subtropical species |
|  | Nudipleura | Doridida | Doridoidea | Discodorididae | *Rostanga orientalis* Rudman & Avern, 1989 | Temperate species |
|  | Nudipleura | Doridida | Doridoidea | Dorididae | *Doriopsis pecten* (Collingwood, 1881) | Tropical–subtropical species |
|  | Nudipleura | Doridida | Doridoidea | Discodorididae | *Discodorididae* sp. 1 | Tropical–subtropical species |
|  | Nudipleura | Doridida | Phyllidioidea | Dendrodorididae | *Dendrodoris arborescens* (Collingwood, 1881) | Tropical–subtropical–temperate species |
|  | Nudipleura | Doridida | Polyceroidea | Polyceridae | *Gymnodoris citrina* (Bergh, 1877) | Tropical–subtropical–temperate species |
|  | Nudipleura | Doridida | Polyceroidea | Polyceridae | *Vayssierea felis* (Collingwood, 1881) | Tropical–subtropical species |
|  | Nudipleura | Nudibranchia | Arminoidea | Arminidae | *Dermatobranchus otome* Baba, 1992 | Temperate species |
|  | Nudipleura | Nudibranchia | Fionoidea | Trinchesiidae | *Tenellia ornata*(Baba, 1937) | Tropical–subtropical species |
| Oodatagamiiwa |  | Aplysiida | Aplysioidea | Aplysiidae | *Aplysia japonica* G. B. Sowerby I, 1869 | Temperate species |
|  |  | Aplysiida | Aplysioidea | Aplysiidae | *Aplysia kurodai* (Baba, 1938) | Tropical–subtropical species |
|  |  | Aplysiida | Aplysioidea | Aplysiidae | *Dolabella auricularia* ([Lightfoot], 1786) | Tropical–subtropical species |
|  | Sacoglossa |  | Plakobranchoidea | Limapontiidae | *Placida dendritica* (Alder & Hancock, 1843) | Temperate species |
|  | Sacoglossa |  | Plakobranchoidea | Limapontiidae | *Ercolania boodleae* (Baba, 1938) | Subarctic–arctic species |
|  | Sacoglossa |  | Plakobranchoidea | Plakobranchidae | *Elysia atroviridis* Baba, 1955 | Temperate species |
|  | Sacoglossa |  | Plakobranchoidea | Plakobranchidae | *Elysia sugashimae* Baba, 1955 | Temperate species |
|  | Nudipleura | Doridida | Chromodoridoidea | Chromodorididae | *Chromodoris orientalis* Rudman, 1983 | Tropical–subtropical species |
|  | Nudipleura | Doridida | Chromodoridoidea | Chromodorididae | *Hypselodoris festiva* (A.Adams, 1861) | Temperate species |
|  | Nudipleura | Doridida | Onchidoridoidea | Goniodorididae | *Pelagella joubini* (Risbec, 1928) | Tropical–subtropical species |
|  | Nudipleura | Doridida | Phyllidioidea | Dendrodorididae | *Doriopsilla miniata* (Alder & Hancock, 1864) | Tropical–subtropical species |
|  | Nudipleura | Doridida | Polyceroidea | Polyceridae | *Vayssierea felis* (Collingwood, 1881) | Tropical–subtropical species |
|  | Nudipleura | Nudibranchia | Arminoidea | Arminidae | *Dermatobranchus otome* Baba, 1992 | Temperate species |
|  | Nudipleura | Nudibranchia | Fionoidea |  | *Tenellia anulata* (Baba, 1949) | Tropical–subtropical species |
| Tanokojima |  | Cephalaspidea | Haminoeoidea | Haminoeidae | *Haloa japonica* (Pilsbry, 1895) | Tropical–subtropical–temperate species |
|  |  | Cephalaspidea | Philinoidea | Gastropteridae | *Siphopteron flavum* (Tokioka & Baba, 1964) | Tropical–subtropical–temperate species |
|  |  | Aplysiida | Aplysioidea | Aplysiidae | *Dolabella auricularia* ([Lightfoot], 1786) | Tropical–subtropical species |
|  |  | Aplysiida | Aplysioidea | Aplysiidae | *Aplysia japonica* G. B. Sowerby I, 1869 | Temperate species |
|  |  | Aplysiida | Aplysioidea | Aplysiidae | *Aplysia kurodai* (Baba, 1937) | Tropical–subtropical species |
|  |  | Aplysiida | Aplysioidea | Aplysiidae | *Petalifera punctulata* (Tapparone Canefri, 1874) | Tropical–subtropical–temperate species |
|  |  | Aplysiida | Aplysioidea | Aplysiidae | *Dolabrifera dolabrifera* (Rang, 1828) | Tropical–subtropical species |
|  | Sacoglossa |  | Plakobranchoidea | Hermaeidae | Aplysiopsis nigra (Baba, 1949) | Temperate species |
|  | Sacoglossa |  | Plakobranchoidea | Limapontiidae | *Placida kevinleei* McCarthy, Krug & Á. Valdés, 2017 | Tropical–subtropical–temperate species |
|  | Sacoglossa |  | Plakobranchoidea | Limapontiidae | *Placida dendritica* (Alder & Hancock, 1843) | Temperate species |
|  | Sacoglossa |  | Plakobranchoidea | Limapontiidae | *Ercolania boodleae* (Baba, 1938) | Subarctic–arctic species |
|  | Sacoglossa |  | Plakobranchoidea | Plakobranchidae | *Elysia amakusana* Baba, 1955 | Tropical–subtropical species |
|  | Sacoglossa |  | Plakobranchoidea | Plakobranchidae | *Elysia obtusa* Baba, 1938 | Tropical–subtropical–temperate species |
|  | Sacoglossa |  | Plakobranchoidea | Plakobranchidae | *Elysia atroviridis* Baba, 1955 | Temperate species |
|  | Sacoglossa |  | Plakobranchoidea | Plakobranchidae | *Elysia nigrocapitata* Baba, 1957 | Temperate species |
|  | Sacoglossa |  | Plakobranchoidea | Plakobranchidae | *Elysia trisinuata* Baba, 1949 | Tropical–subtropical species |
|  | Sacoglossa |  | Plakobranchoidea | Plakobranchidae | *Elysia sugashimae* Baba, 1955 | Temperate species |
|  | Sacoglossa |  | Plakobranchoidea | Plakobranchidae | *Elysia ornata* (Swainson, 1840) | Tropical–subtropical–temperate species |
|  | Nudipleura | Doridida | Chromodoridoidea | Chromodorididae | *Chromodoris orientalis* Rudman, 1983 | Tropical–subtropical species |
|  | Nudipleura | Doridida | Chromodoridoidea | Chromodorididae | *Hypselodoris festiva* (A.Adams, 1861) | Temperate species |
|  | Nudipleura | Doridida | Chromodoridoidea | Chromodorididae | *Goniobranchus tinctorius* (Rüppell & Leuckart, 1830) | Tropical–subtropical species |
|  | Nudipleura | Doridida | Chromodoridoidea | Chromodorididae | *Verconia nivalis* (Baba, 1937) | Temperate species |
|  | Nudipleura | Doridida | Chromodoridoidea | Chromodorididae | *Goniobranchus tumuliferus* (Collingwood, 1881) | Tropical–subtropical species |
|  | Nudipleura | Doridida | Doridoidea | Discodorididae | *Rostanga orientalis* Rudman & Avern, 1989 | Temperate species |
|  | Nudipleura | Doridida | Doridoidea | Dorididae | *Doriopsis pecten* (Collingwood, 1881) | Tropical–subtropical species |
|  | Nudipleura | Doridida | Doridoidea | Ddiscodorididae | *Discodorididae* sp. 1 | Tropical–subtropical species |
|  | Nudipleura | Doridida | Doridoidea | Discodorididae | *Jorunna parva* (Baba, 1938) | Tropical–subtropical species |
|  | Nudipleura | Doridida | Doridoidea | Dorididae | *Homoiodoris japonica* Bergh, 1882 | Temperate species |
|  | Nudipleura | Doridida | Doridoidea | Discodorididae | *Platydoris ellioti* (Alder & Hancock, 1864) | Tropical–subtropical species |
|  | Nudipleura | Doridida | Onchidoridoidea | Goniodorididae | *Bermudella distincta* (Baba, 1940) | Temperate species |
|  | Nudipleura | Doridida | Onchidoridoidea | Goniodorididae | *Pelagella joubini* (Risbec, 1928) | Tropical–subtropical species |
|  | Nudipleura | Doridida | Onchidoridoidea | Goniodorididae | *Pelagella castanea* (Alder & Hancock, 1845) | Tropical–subtropical–temperate species |
|  | Nudipleura | Doridida | Onchidoridoidea | Goniodorididae | *Ceratodoris hiroi*(Baba, 1938) | Tropical–subtropical species |
|  | Nudipleura | Doridida | Polyceroidea | Polyceridae | *Polycera fujitai* Baba, 1937 | Tropical–subtropical species |
|  | Nudipleura | Doridida | Polyceroidea | Polyceridae | *Palio amakusana* Baba, 1960 | Temperate species |
|  | Nudipleura | Doridida | Polyceroidea | Polyceridae | *Gymnodoris inornata* (Bergh, 1880) | Tropical–subtropical species |
|  | Nudipleura | Doridida | Polyceroidea | Polyceridae | *Gymnodoris subornata* Baba, 1960 | Temperate species |
|  | Nudipleura | Doridida | Polyceroidea | Polyceridae | *Gymnodoris citrina* (Bergh, 1877) | Temperate species |
|  | Nudipleura | Doridida | Polyceroidea | Polyceridae | *Kaloplocamus ramosus* (Cantraine, 1835) | Tropical–subtropical–temperate species |
|  | Nudipleura | Doridida | Polyceroidea | Polyceridae | *Kaloplocamus acutus* Baba, 1949 | Tropical–subtropical species |
|  | Nudipleura | Doridida | Polyceroidea | Polyceridae | *Vayssierea felis* (Collingwood, 1881) | Tropical–subtropical species |
|  | Nudipleura | Doridida | Phyllidioidea | Dendrodorididae | *Dendrodoris arborescens* (Collingwood, 1881) | Tropical–subtropical–temperate species |
|  | Nudipleura | Doridida | Phyllidioidea | Dendrodorididae | *Dendrodoris fumata* (Rüppell & Leuckart, 1830) | Tropical–subtropical–temperate species |
|  | Nudipleura | Doridida | Phyllidioidea | Dendrodorididae | *Dendrodoris krusensternii* (J. E. Gray, 1850) | Tropical–subtropical–temperate species |
|  | Nudipleura | Doridida | Phyllidioidea | Dendrodorididae | *Dendrodoris guttata* (Odhner, 1917) | Tropical–subtropical species |
|  | Nudipleura | Nudibranchia | Aeolidioidea | Facelinidae | *Cratena lineata* (Eliot, 1905) | Tropical–subtropical species |
|  | Nudipleura | Nudibranchia | Aeolidioidea | Facelinidae | *Herviella affinis* Baba, 1960 | Temperate species |
|  | Nudipleura | Nudibranchia | Aeolidioidea | Facelinidae | *Herviella yatsui* (Baba, 1930) | Tropical–subtropical species |
|  | Nudipleura | Nudibranchia | Aeolidioidea | Facelinidae | *Sakuraeolis enosimensis* (Baba, 1930) | Temperate species |
|  | Nudipleura | Nudibranchia | Aeolidioidea | Aeolidiidae | *Anteaeolidiella takanosimensis* (Baba, 1930) | Temperate species |
|  | Nudipleura | Nudibranchia | Aeolidioidea | Aeolidiidae | *Limenandra fusiformis* (Baba, 1949) | Tropical–subtropical species |
|  | Nudipleura | Nudibranchia | Aeolidioidea | Aeolidiidae | *Baeolidia japonica* Baba, 1933 | Temperate species |
|  | Nudipleura | Nudibranchia | Arminoidea | Arminidae | *Dermatobranchus otome* Baba, 1992 | Temperate species |
|  | Nudipleura | Nudibranchia | Arminoidea | Arminidae | *Dermatobranchus striatellus* Baba, 1949 | Tropical–subtropical species |
|  | Nudipleura | Nudibranchia | Dendronotoidea | Bornellidae | *Bornella stellifera* (A. Adams & Reeve, 1848) | Tropical–subtropical species |
|  | Nudipleura | Nudibranchia | Dendronotoidea | Dotidae | *Doto japonica* Odhner, 1936 | Temperate species |
|  | Nudipleura | Nudibranchia | Dendronotoidea | Dotidae | *Doto bella* Baba, 1938 | Temperate species |
|  | Nudipleura | Nudibranchia | Fionoidea | Eubranchidae | *Eubranchopsis virginalis* Baba, 1949 | Tropical–subtropical species |
|  | Nudipleura | Nudibranchia | Fionoidea | Eubranchidae | *Eubranchus inabai* Baba, 1964 | Tropical–subtropical species |
|  | Nudipleura | Nudibranchia | Fionoidea | Eubranchidae | *Eubranchus horii* Baba, 1960 | Temperate species |
|  | Nudipleura | Nudibranchia | Fionoidea | Eubranchidae | *Leostyletus misakiensis* (Baba, 1960) | Subarctic–arctic species |
|  | Nudipleura | Nudibranchia | Fionoidea | Cuthonidae | *Cuthona futairo* Baba, 1963 | Temperate species |
|  | Nudipleura | Nudibranchia | Fionoidea | Trinchesiidae | *Catriona pinnifera* (Baba, 1949) | Tropical–subtropical species |
|  | Nudipleura | Nudibranchia | Fionoidea | Trinchesiidae | *Tenellia ornata* (Baba, 1937) | Tropical–subtropical species |
|  | Nudipleura | Nudibranchia | Fionoidea | Trinchesiidae | *Tenellia pupillae* (Baba, 1961) | Temperate species |
|  | Nudipleura | Pleurobranchida | Pleurobranchoidea | Pleurobranchidae | *Pleurehdera stellata* (Risso, 1826) | Tropical–subtropical–temperate species |
|  | Nudipleura | Pleurobranchida | Pleurobranchoidea | Pleurobranchaeidae | *Pleurobranchaea maculata* (Quoy & Gaimard, 1832) | Tropical–subtropical species |
| Takahama Ingeri-bana Cape | Sacoglossa |  | Plakobranchoidea | Hermaeidae | Aplysiopsis nigra (Baba, 1949) | Temperate species |
|  | Sacoglossa |  | Plakobranchoidea | Limapontiidae | *Ercolania boodleae* (Baba, 1938) | Subarctic–arctic species |
|  | Sacoglossa |  | Plakobranchoidea | Limapontiidae | *Placida dendritica* (Alder & Hancock, 1843) | Temperate species |
|  | Sacoglossa |  | Plakobranchoidea | Plakobranchidae | *Elysia atroviridis* Baba, 1955 | Temperate species |
|  | Sacoglossa |  | Plakobranchoidea | Plakobranchidae | *Elysia amakusana* Baba, 1955 | Tropical–subtropical species |
| Igeshuku,MeotoIwa | Sacoglossa |  | Oxynooidea | Oxynoidae | *Oxynoe viridis* (Pease, 1861) | Tropical–subtropical–temperate species |
|  | Sacoglossa |  | Plakobranchoidea | Limapontiidae | *Placida kevinleei* McCarthy, Krug & Á. Valdés, 2017 | Tropical–subtropical–temperate species |
|  | Sacoglossa |  | Plakobranchoidea | Plakobranchidae | *Elysia obtusa* Baba, 1938 | Tropical–subtropical–temperate species |

1. From Kawahara (unpublished).

| **Site** | **Date** | **Superorder** | **Order** | **Superfamily** | **Family** | **Species** | **Climate classification** | **Number of individuals** |
| --- | --- | --- | --- | --- | --- | --- | --- | --- |
| Tatsunokuchi | 2001-05-04 | Nudipleura | Doridida | Chromodoridoidea | Chromodorididae | *Doriprismatica atromarginata* (Cuvier, 1804) | Tropical–subtropical–temperate species | 1 |
| Tatsunokuchi |  | Nudipleura | Doridida | Chromodoridoidea | Chromodorididae | *Hypselodoris festiva* (A.Adams, 1861) | Temperate species | 1 |
| Tatsunokuchi |  | Nudipleura | Doridida | Chromodoridoidea | Chromodorididae | *Verconia subnivalis*(Baba, 1987) | Temperate species | 1 |
| Tatsunokuchi |  | Nudipleura | Doridida | Doridoidea | Discodorididae | *Jorunna parva*(Baba, 1938) | Tropical–subtropical species | 1 |
| Tatsunokuchi |  | Nudipleura | Doridida | Phyllidioidea | Dendrodorididae | *Dendrodoris krusensternii* (J. E. Gray, 1850) | Tropical–subtropical–temperate species | 1 |
| Tatsunokuchi |  | Nudipleura | Nudibranchia | Arminoidea | Arminidae | *Dermatobranchus primus Baba*, 1976 | Temperate species | 1 |
| Tatsunokuchi |  | Nudipleura | Nudibranchia | Fionoidea | Trinchesiidae | *Tenellia diversicolor*(Baba, 1975) | Tropical–subtropical species | 1 |
| Tatsunokuchi | 2001-06-24 | Sacoglossa |  | Plakobranchoidea | Plakobranchidae | *Elysia atroviridis Baba*, 1955 | Temperate species | 2 |
| Tatsunokuchi |  | Nudipleura | Doridida | Chromodoridoidea | Chromodorididae | *Chromodoris orientalis* Rudman, 1983 | Tropical–subtropical species | 1 |
| Tatsunokuchi | 2001-06-30 | Nudipleura | Doridida | Chromodoridoidea | Chromodorididae | *Chromodoris orientalis* Rudman, 1983 | Tropical–subtropical species | 2 |
| Tatsunokuchi |  | Nudipleura | Doridida | Chromodoridoidea | Chromodorididae | *Verconia purpurea*(Baba, 1949) | Temperate species | 1 |
| Tatsunokuchi |  | Nudipleura | Doridida | Chromodoridoidea | Chromodorididae | *Verconia norba*(Er. Marcus & Ev. Marcus, 1970) | Tropical–subtropical–temperate species | 1 |
| Tatsunokuchi |  | Nudipleura | Doridida | Phyllidioidea | Phyllidiidae | *Phyllidiella pustulosa* (Cuvier, 1804) | Tropical–subtropical–temperate species | 1 |
| Tatsunokuchi | 2001-07-05 | Nudipleura | Doridida | Chromodoridoidea | Chromodorididae | *Chromodoris orientalis* Rudman, 1983 | Tropical–subtropical species | 3 |
| Tatsunokuchi |  | Nudipleura | Doridida | Chromodoridoidea | Chromodorididae | *Doriprismatica atromarginata* (Cuvier, 1804) | Tropical–subtropical–temperate species | 4 |
| Tatsunokuchi |  | Nudipleura | Doridida | Chromodoridoidea | Chromodorididae | *Hypselodoris sagamiensis* (Baba, 1949) | Tropical–subtropical species | 3 |
| Tatsunokuchi |  | Nudipleura | Doridida | Chromodoridoidea | Chromodorididae | *Verconia purpurea*(Baba, 1949) | Temperate species | 4 |
| Tatsunokuchi |  | Nudipleura | Doridida | Phyllidioidea | Phyllidiidae | *Phyllidiella pustulosa* (Cuvier, 1804) | Tropical–subtropical–temperate species | 3 |
| Tatsunokuchi | 2001-07-20 | Nudipleura | Doridida | Polyceroidea | Polyceridae | *Gymnodoris impudica*(Rüppell & Leuckart, 1830) | Tropical–subtropical species | 1 |
| Tatsunokuchi |  | Nudipleura | Doridida | Chromodoridoidea | Chromodorididae | *Doriprismatica atromarginata* (Cuvier, 1804) | Tropical–subtropical–temperate species | 3 |
| Tatsunokuchi |  | Nudipleura | Doridida | Chromodoridoidea | Chromodorididae | *Hypselodoris festiva* (A.Adams, 1861) | Temperate species | 1 |
| Tatsunokuchi | 2001-08-02 | Nudipleura | Doridida | Chromodoridoidea | Chromodorididae | *Doriprismatica atromarginata* (Cuvier, 1804) | Tropical–subtropical–temperate species | 1 |
| Tatsunokuchi | 2001-10-02 |  |  |  |  | None observed |  |  |
| Tatsunokuchi | 2001-12-15 |  |  |  |  | None observed |  |  |
| Tatsunokuchi | 2002-04-26 |  | Aplysiida | Aplysioidea | Aplysiidae | *Aplysia kurodai* (Baba, 1937) | Tropical–subtropical species | 12 |
| Tatsunokuchi | 2002-04-29 |  | Aplysiida | Aplysioidea | Aplysiidae | *Aplysia kurodai* (Baba, 1938) | Tropical–subtropical species | 117 |
| Tatsunokuchi |  |  | Aplysiida | Aplysioidea | Aplysiidae | *Aplysia japonica* G. B. Sowerby I, 1869 | Temperate species | 1 |
| Tatsunokuchi |  | Nudipleura | Doridida | Onchidoridoidea | Goniodorididae | *Ceratodoris hiroi*(Baba, 1938) | Tropical–subtropical species | 1 |
| Tatsunokuchi | 2002-05-13 |  | Aplysiida | Aplysioidea | Aplysiidae | *Aplysia kurodai* (Baba, 1937) | Tropical–subtropical species | 50 |
| Tatsunokuchi |  | Sacoglossa |  | Plakobranchoidea | Limapontiidae | *Placida dendritica* (Alder & Hancock, 1843) | Temperate species | 1 |
| Tatsunokuchi |  | Sacoglossa |  | Plakobranchoidea | Plakobranchidae | *Elysia trisinuata* Baba, 1949 | Tropical–subtropical species | 1 |
| Tatsunokuchi |  | Nudipleura | Doridida | Chromodoridoidea | Chromodorididae | *Chromodoris orientalis* Rudman, 1983 | Tropical–subtropical species | 1 |
| Tatsunokuchi |  | Nudipleura | Doridida | Chromodoridoidea | Chromodorididae | *Goniobranchus tinctorius* (Rüppell & Leuckart, 1830) | Tropical–subtropical species | 1 |
| Tatsunokuchi |  | Nudipleura | Doridida | Chromodoridoidea | Chromodorididae | *Hypselodoris festiva* (A.Adams, 1861) | Temperate species | 1 |
| Tatsunokuchi | 2002-06-05 | Nudipleura | Doridida | Onchidoridoidea | Goniodorididae | *Ceratodoris hiroi*(Baba, 1938) | Tropical–subtropical species | 5 |
| Tatsunokuchi |  | Nudipleura | Doridida | Phyllidioidea | Dendrodorididae | *Dendrodoris krusensternii* (J. E. Gray, 1850) | Tropical–subtropical–temperate species | 1 |
| Tatsunokuchi | 2002-06-28 | Sacoglossa |  | Plakobranchoidea | Plakobranchidae | *Elysia trisinuata* Baba, 1949 | Tropical–subtropical species | 1 |
| Tatsunokuchi |  | Nudipleura | Doridida | Chromodoridoidea | Chromodorididae | *Hypselodoris festiva* (A.Adams, 1861) | Temperate species | 1 |
| Tatsunokuchi | 2002-07-12 | Sacoglossa |  | Plakobranchoidea | Plakobranchidae | *Elysia trisinuata* Baba, 1949 | Tropical–subtropical species | 2 |
| Tatsunokuchi | 2002-08-06 |  |  |  |  | None observed |  |  |
| Tatsunokuchi | 2002-08-15 |  |  |  |  | None observed |  |  |
| Tatsunokuchi | 2002-09-18 | Sacoglossa |  | Plakobranchoidea | Plakobranchidae | *Elysia ornata* (Swainson, 1840) | Tropical–subtropical–temperate species | 1 |
| Tatsunokuchi | 2002-09-24 | Nudipleura | Doridida | Chromodoridoidea | Chromodorididae | *Doriprismatica atromarginata* (Cuvier, 1804) | Tropical–subtropical–temperate species | 1 |
| Tatsunokuchi |  | Nudipleura | Doridida | Phyllidioidea | Dendrodorididae | *Dendrodoris krusensternii* (J. E. Gray, 1850) | Tropical–subtropical–temperate species | 1 |
| Tatsunokuchi | 2002-10-09 |  |  |  |  | None observed |  |  |
| Tatsunokuchi | 2002-10-17 |  |  |  |  | None observed |  |  |
| Tatsunokuchi | 2002-11-28 | Sacoglossa |  | Plakobranchoidea | Plakobranchidae | *Elysia ornata* (Swainson, 1840) | Tropical–subtropical–temperate species | 1 |
| Tatsunokuchi |  | Nudipleura | Doridida | Polyceroidea | Polyceridae | *Polycera japonica Baba*, 1949 | Tropical–subtropical–temperate species | 1 |
| Tatsunokuchi | 2002-12-06 | Sacoglossa |  | Plakobranchoidea | Limapontiidae | *Placida kevinleei McCarthy*, Krug & Á. Valdés, 2017 | Tropical–subtropical–temperate species | 9 |
| Tatsunokuchi |  | Nudipleura | Doridida | Phyllidioidea | Phyllidiidae | *Phyllidiella pustulosa* (Cuvier, 1804) | Tropical–subtropical–temperate species | 1 |
| Tatsunokuchi | 2003-03-29 | Sacoglossa |  | Plakobranchoidea | Limapontiidae | *Placida dendritica* (Alder & Hancock, 1843) | Temperate species | 1 |
| Tatsunokuchi | 2003-03-30 |  | Aplysiida | Aplysioidea | Aplysiidae | *Aplysia kurodai* (Baba, 1937) | Tropical–subtropical species | 1 |
| Tatsunokuchi |  | Sacoglossa |  | Plakobranchoidea | Hermaeidae | *Aplysiopsis minor*(Baba, 1959) | Temperate species | 4 |
| Tatsunokuchi |  | Sacoglossa |  | Plakobranchoidea | Limapontiidae | *Placida dendritica* (Alder & Hancock, 1843) | Temperate species | 14 |
| Tatsunokuchi |  | Sacoglossa |  | Plakobranchoidea | Limapontiidae | *Ercolania boodleae*(Baba, 1938) | Tropical–subtropical species | 24 |
| Tatsunokuchi |  | Sacoglossa |  | Plakobranchoidea | Plakobranchidae | *Elysia atroviridis Baba*, 1955 | Temperate species | 3 |
| Tatsunokuchi | 2003-05-21 |  | Aplysiida | Aplysioidea | Aplysiidae | *Aplysia kurodai* (Baba, 1937) | Tropical–subtropical species | 2 |
| Tatsunokuchi | 2003-06-03 | Nudipleura | Doridida | Chromodoridoidea | Chromodorididae | *Chromodoris orientalis* Rudman, 1983 | Tropical–subtropical species | 1 |
| Tatsunokuchi | 2003-07-23 | Nudipleura | Doridida | Chromodoridoidea | Chromodorididae | *Doriprismatica atromarginata* (Cuvier, 1804) | Tropical–subtropical–temperate species | 1 |
| Tatsunokuchi | 2003-09-24 | Sacoglossa |  | Plakobranchoidea | Plakobranchidae | *Elysia ornata* (Swainson, 1840) | Tropical–subtropical–temperate species | 8 |
| Tatsunokuchi |  | Nudipleura | Doridida | Chromodoridoidea | Chromodorididae | *Doriprismatica atromarginata* (Cuvier, 1804) | Tropical–subtropical–temperate species | 3 |
| Nomozaki-Akase | 2001-05-05 | Nudipleura | Doridida | Chromodoridoidea | Chromodorididae | *Chromodoris orientalis* Rudman, 1983 | Tropical–subtropical species | 3 |
| Nomozaki-Akase |  | Nudipleura | Doridida | Chromodoridoidea | Chromodorididae | *Goniobranchus tinctorius* (Rüppell & Leuckart, 1830) | Tropical–subtropical species | 1 |
| Nomozaki-Akase |  | Nudipleura | Doridida | Chromodoridoidea | Chromodorididae | *Hypselodoris festiva* (A.Adams, 1861) | Temperate species | 6 |
| Nomozaki-Akase |  | Nudipleura | Doridida | Onchidoridoidea | Goniodorididae | *Ceratodoris hiroi*(Baba, 1938) | Tropical–subtropical species | 1 |
| Nomozaki-Akase |  | Nudipleura | Doridida | Phyllidioidea | Dendrodorididae | *Dendrodoris krusensternii* (J. E. Gray, 1850) | Tropical–subtropical–temperate species | 1 |
| Nomozaki-Akase | 2001-06-09 | Nudipleura | Doridida | Chromodoridoidea | Chromodorididae | *Chromodoris orientalis* Rudman, 1983 | Tropical–subtropical species | 1 |
| Nomozaki-Akase |  | Nudipleura | Doridida | Phyllidioidea | Dendrodorididae | *Dendrodoris krusensternii* (J. E. Gray, 1850) | Tropical–subtropical–temperate species | 3 |
| Nomozaki-Akase |  | Nudipleura | Nudibranchia | Arminoidea | Arminidae | *Dermatobranchus striatellus Baba*, 1949 | Tropical–subtropical species | 3 |
| Nomozaki-Akase | 2001-07-31 | Nudipleura | Doridida | Chromodoridoidea | Chromodorididae | *Hypselodoris festiva* (A.Adams, 1861) | Temperate species | 1 |
| Nomozaki-Akase |  | Nudipleura | Doridida | Chromodoridoidea | Chromodorididae | *Doriprismatica atromarginata* (Cuvier, 1804) | Tropical–subtropical–temperate species | 1 |
| Nomozaki-Akase |  | Nudipleura | Doridida | Phyllidioidea | Phyllidiidae | *Phyllidiella pustulosa* (Cuvier, 1804) | Tropical–subtropical–temperate species | 1 |
| Nomozaki-Akase |  | Nudipleura | Nudibranchia | Tritonioidea | Tritoniidae | *Tritoniopsis elegans* (Audouin, 1826) | Tropical–subtropical species | 1 |
| Nomozaki-Akase | 2001-08-04 |  | Aplysiida | Aplysioidea | Aplysiidae | *Aplysia kurodai* (Baba, 1937) | Tropical–subtropical species | 4 |
| Nomozaki-Akase |  | Nudipleura | Doridida | Chromodoridoidea | Chromodorididae | *Chromodoris orientalis* Rudman, 1983 | Tropical–subtropical species | 4 |
| Nomozaki-Akase |  | Nudipleura | Doridida | Chromodoridoidea | Chromodorididae | *Goniobranchus fidelis* (Kelaart, 1858) | Tropical–subtropical–temperate species | 2 |
| Nomozaki-Akase |  | Nudipleura | Doridida | Chromodoridoidea | Chromodorididae | *Goniobranchus sinensis* (Rudman, 1985) | Tropical–subtropical species | 1 |
| Nomozaki-Akase |  | Nudipleura | Doridida | Phyllidioidea | Dendrodorididae | *Dendrodoris guttata*(Odhner, 1917) | Tropical–subtropical species | 2 |
| Nomozaki-Akase | 2001-08-08 | Nudipleura | Doridida | Chromodoridoidea | Actinocyclidae | *Actinocyclus papillatus*(Bergh, 1878) | Tropical–subtropical species | 1 |
| Nomozaki-Akase | 2001-08-31 | Nudipleura | Nudibranchia | Samloidea | Samlidae | *Samla takashigei* Korshunova, Martynov, Bakken, Evertsen, Fletcher, Mudianta, H. Saito, Lundin, Schrödl & Picton, 2017 | Tropical–subtropical species | 5 |
| Nomozaki-Akase | 2001-09-02 | Nudipleura | Doridida | Phyllidioidea | Dendrodorididae | *Dendrodoris krusensternii* (J. E. Gray, 1850) | Tropical–subtropical–temperate species | 3 |
| Nomozaki-Akase | 2001-09-04 | Nudipleura | Nudibranchia | Samloidea | Samlidae | *Samla takashigei* Korshunova, Martynov, Bakken, Evertsen, Fletcher, Mudianta, H. Saito, Lundin, Schrödl & Picton, 2017 | Tropical–subtropical species | 3 |
| Nomozaki-Akase | 2001-10-10 |  |  |  |  | None observed |  |  |
| Nomozaki-Akase | 2002-04-03 |  | Cephalaspidea | Haminoeoidea | Haminoeidae | *Haloa japonica*(Pilsbry, 1895) | Tropical–subtropical–temperate species | 2 |
| Nomozaki-Akase |  |  | Aplysiida | Aplysioidea | Aplysiidae | *Aplysia oculifera A*. Adams & Reeve, 1850 | Tropical–subtropical species | 12 |
| Nomozaki-Akase |  |  | Aplysiida | Aplysioidea | Aplysiidae | *Aplysia japonica* G. B. Sowerby I, 1869 | Temperate species | 1 |
| Nomozaki-Akase |  | Nudipleura | Nudibranchia | Aeolidioidea | Facelinidae | *Sakuraeolis enosimensis*(Baba, 1930) | Temperate species | 1 |
| Nomozaki-Akase | 2002-05-02 |  | Aplysiida | Aplysioidea | Aplysiidae | *Aplysia japonica* G. B. Sowerby I, 1869 | Temperate species | 2 |
| Nomozaki-Akase | 2002-05-04 |  | Aplysiida | Aplysioidea | Aplysiidae | *Aplysia kurodai* (Baba, 1937) | Tropical–subtropical species | 50 |
| Nomozaki-Akase |  |  | Aplysiida | Aplysioidea | Aplysiidae | *Aplysia oculifera A*. Adams & Reeve, 1850 | Tropical–subtropical species | 50 |
| Nomozaki-Akase |  | Nudipleura | Doridida | Chromodoridoidea | Chromodorididae | *Chromodoris orientalis* Rudman, 1983 | Tropical–subtropical species | 2 |
| Nomozaki-Akase |  | Nudipleura | Doridida | Doridoidea | Discodorididae | *Rostanga orientalis Rudman* & Avern, 1989 | Temperate species | 1 |
| Nomozaki-Akase |  | Nudipleura | Doridida | Doridoidea | Discodorididae | *Jorunna parva*(Baba, 1938) | Tropical–subtropical species | 1 |
| Nomozaki-Akase | 2002-06-01 | Nudipleura | Doridida | Chromodoridoidea | Chromodorididae | *Chromodoris orientalis* Rudman, 1983 | Tropical–subtropical species | 1 |
| Nomozaki-Akase | 2002-08-27 | Sacoglossa |  | Plakobranchoidea | Plakobranchidae | *Elysia ornata* (Swainson, 1840) | Tropical–subtropical–temperate species | 14 |
| Nomozaki-Akase |  | Nudipleura | Doridida | Phyllidioidea | Dendrodorididae | *Dendrodoris krusensternii* (J. E. Gray, 1850) | Tropical–subtropical–temperate species | 1 |
| Nomozaki-Akase |  | Nudipleura | Nudibranchia | Samloidea | Samlidae | *Samla takashigei* Korshunova, Martynov, Bakken, Evertsen, Fletcher, Mudianta, H. Saito, Lundin, Schrödl & Picton, 2017 | Tropical–subtropical species | 5 |
| Nomozaki-Akase | 2002-10-24 |  |  |  |  | None observed |  |  |
