# Supplementary material for: A different world: temporal changes in the community structure of sea slugs (Heterobranchia) in northwest Japan spanning more than a half-century
Source: PeerJ. 2026 Mar 2;14:e20870. doi: 10.7717/peerj.20870 (PMC12962135; doi:10.7717/peerj.20870)
Supplement: Supplemental Information 5 [file peerj-14-20870-s005.docx]

| **Survey Date** | **Site** | **Method** | **Start** | **End** | **Duration**  **（min）** | **Air**  **(℃)** | **Water**  **(℃)** | **Mean Depth**  **（ｍ）** | **Max Depth**  **（ｍ）** | **Visibility**  **(m)** | **Tide** | **Sea Conditions** | **Weather** |
| --- | --- | --- | --- | --- | --- | --- | --- | --- | --- | --- | --- | --- | --- |
| 2023-06-02 | Tatsunokuchi | Scuba diving | 11:39 | 12:46 | 67 | 18 | 20.8 | 3.6 | 5.5 | 5 | Medium tide | Calm | Rainy |
| 2023-07-05 | Nomozaki-Akase | Scuba diving | 9:54 | 11:15 | 81 | 25 | 23.8 | 4.0 | 7.2 | 8 | Medium tide  (at low tide) | Calm | Rainy |
| 2023-07-05 | Nomozaki-Akase | Scuba diving | 11:51 | 12:50 | 51 | 27 | 23.8 | 2.9 | 5.3 | 8 | Medium tide  (at low tide) | Calm | Rainy |
| 2023-07-19 | Nomozaki-Akase | Scuba diving | 9:19 | 10:40 | 81 | 26 | 25.9 | 3.6 | 5.3 | 8 | Medium tide  (at low tide) | Slightly wavy | Rainy |
| 2023-07-19 | Nomozaki-Akase | Scuba diving | 11:11 | 12:22 | 72 | 27 | 25.9 | 4.1 | 7.4 | 8 | Spring tide  (ebb tide) | Slightly wavy | Rainy |
| 2023-07-31 | Tatsunokuchi | Scuba diving | 9:01 | 9:56 | 55 | 35 | 28.6 | 4.2 | 6.2 | 5 | Neap tide | Calm | Rainy |
| 2023-07-31 | Tatsunokuchi | Scuba diving | 12:06 | 13:02 | 56 | 35 | 27.2 | 6.9 | 14.3 | 5 | Neap tide | Calm | Rainy |
| 2023-08-02 | Nomozaki-Akase | Scuba diving | 9:28 | 10:34 | 66 | 33 | 26.5 | 3.9 | 7.9 | 8 | Spring tide  (ebb tide) | Wavy | Sunny |
| 2023-08-02 | Nomozaki-Akase | Scuba diving | 11:07 | 12:09 | 62 | 33 | 27.2 | 2.3 | 3.8 | 8 | Spring tide  (ebb tide) | Wavy | Sunny |
| 2023-08-29 | Tatsunokuchi | Scuba diving | 8:59 | 9:56 | 59 | 31 | 25.8 | 7.1 | 13.6 | 3 | Spring tide  (ebb tide) | Calm | Sunny |
| 2023-08-29 | Tatsunokuchi | Scuba diving | 10:53 | 11:48 | 55 | 31 | 27.6 | 3.5 | 5.5 | 3 | Spring tide (  ebb tide) | Calm | Cloudy |
| 2023-09-19 | Tatsunokuchi | Scuba diving | 9:37 | 10:38 | 61 | 26 | 27.7 | 7.1 | 10.8 | 5 | Spring tide | Calm | Cloudy |
| 2023-09-19 | Tatsunokuchi | Scuba diving | 11:34 | 12:40 | 66 | 26 | 27.4 | 4.8 | 8.6 | 5 | Spring tide | Calm | Sunny |
| 2023-09-29 | Nomozaki-Akase | Scuba diving | 9:49 | 11:30 | 73 | 25 | 26.9 | 3.5 | 5.7 | 8 | Spring tide | Calm | Sunny |
| 2023-09-29 | Nomozaki-Akase | Scuba diving | 11:43 | 12:31 | 48 | 28 | 27 | 2.7 | 5.2 | 8 | Spring tide | Calm | Sunny |
| 2023-10-03 | Nomozaki-Akase | Scuba diving | 9:30 | 10:26 | 56 | 23 | 25.9 | 3.7 | 5.3 | 8 | Medium tide  (ebb tide) | Calm | Sunny |
| 2023-10-03 | Nomozaki-Akase | Scuba diving | 11:10 | 12:25 | 75 | 25 | 25.8 | 4.4 | 7.4 | 8 | Medium tide  (ebb tide) | Calm | Sunny |
| 2023-10-23 | Tatsunokuchi | Scuba diving | 9:41 | 10:35 | 54 | 20 | 23.5 | 9.6 | 14.3 | 5 | Neap tide | Calm | Sunny |
| 2023-10-23 | Tatsunokuchi | Scuba diving | 11:26 | 12:06 | 40 | 21 | 22.9 | 4.1 | 6.7 | 5 | Neap tide | Calm | Sunny |
| 2023-11-16 | Nomozaki-Akase | Scuba diving | 9:38 | 10:31 | 53 | 13 | 20.4 | 3.3 | 5.5 | 8 | Medium tide  (at high tide) | Calm | Cloudy |
| 2023-11-24 | Tatsunokuchi | Scuba diving | 9:19 | 10:03 | 44 | 13 | 18.4 | 4.5 | 5.7 | 4 | Medium tide  (at low tide) | Calm | Cloudy |
| 2023-01-16 | Nomozaki-Akase | Scuba diving | 9:57 | 10:53 | 56 | 7 | 14.5 | 3.9 | 5.8 | 8 | Medium tide  (at high tide) | Wavy | Sunny |
| 2023-01-16 | Nomozaki-Akase | Scuba diving | 11:45 | 12:42 | 57 | 9 | 15.2 | 3.3 | 4.2 | 8 | Medium tide  (at high tide) | Wavy | Sunny |
| 2024-01-19 | Tatsunokuchi | Scuba diving | 9:19 | 10:26 | 67 | 12 | 15.4 | 12.4 | 19.4 | 5 | Neap tide | Calm | Rainy |
| 2024-01-19 | Tatsunokuchi | Scuba diving | 11:36 | 12:48 | 72 | 14 | 15.2 | 4.9 | 7.7 | 5 | Neap tide | Calm | Cloudy |
