# Supplementary material for: A different world: temporal changes in the community structure of sea slugs (Heterobranchia) in northwest Japan spanning more than a half-century
Source: PeerJ. 2026 Mar 2;14:e20870. doi: 10.7717/peerj.20870 (PMC12962135; doi:10.7717/peerj.20870)
Supplement: Supplemental Information 6 [file peerj-14-20870-s006.docx]

| **Site** | **date** | **Superorder** | **Order** | **Superfamily** | **Family** | **Species** | **Climate classification** | **Number of individuals** |
| --- | --- | --- | --- | --- | --- | --- | --- | --- |
| Nomozaki-Akase | 2023-07-05 |  | Aplysiida | Aplysioidea | Aplysiidae | *Aplysia japonica* G. B. Sowerby I, 1869 | Temperate species | 3 |
| Nomozaki-Akase |  | Sacoglossa |  | Plakobranchoidea | Plakobranchidae | *Elysia trisinuata* Baba, 1949 | Tropical–subtropical species | 3 |
| Nomozaki-Akase |  | Nudipleura | Doridida | Chromodoridoidea | Chromodorididae | *Doriprismatica atromarginata* (Cuvier, 1804) | Tropical–subtropical–temperate species | 2 |
| Nomozaki-Akase |  | Nudipleura | Doridida | Chromodoridoidea | Chromodorididae | *Chromodoris orientalis* Rudman, 1983 | Tropical–subtropical species | 2 |
| Nomozaki-Akase |  | Nudipleura | Doridida | Chromodoridoidea | Chromodorididae | *Goniobranchus sinensis* (Rudman, 1985) | Tropical–subtropical species | 1 |
| Nomozaki-Akase |  | Nudipleura | Doridida | Chromodoridoidea | Chromodorididae | *Goniobranchus tinctorius* (Rüppell & Leuckart, 1830) | Tropical–subtropical species | 2 |
| Nomozaki-Akase |  | Nudipleura | Doridida | Chromodoridoidea | Chromodorididae | *Hypselodoris festiva* (A.Adams, 1861) | Temperate species | 2 |
| Nomozaki-Akase |  | Nudipleura | Doridida | Chromodoridoidea | Chromodorididae | *Hypselodoris placida* (Baba, 1949) | Temperate species | 1 |
| Nomozaki-Akase |  | Nudipleura | Doridida | Phyllidioidea | Dendrodorididae | *Dendrodoris krusensternii* (J. E. Gray, 1850) | Tropical–subtropical–temperate species | 1 |
| Nomozaki-Akase |  | Nudipleura | Nudibranchia | Tritonioidea | Tritoniidae | *Tritoniopsis elegans* (Audouin, 1826) | Tropical–subtropical species | 2 |
| Nomozaki-Akase | 2023-07-19 |  | Aplysiida | Aplysioidea | Aplysiidae | *Aplysia japonica* G. B. Sowerby I, 1869 | Temperate species | 3 |
| Nomozaki-Akase |  | Sacoglossa |  | Plakobranchoidea | Plakobranchidae | *Elysia trisinuata* Baba, 1949 | Tropical–subtropical species | 1 |
| Nomozaki-Akase |  | Sacoglossa |  | Plakobranchoidea | Plakobranchidae | *Thuridilla albopustulosa* Gosliner, 1995 | Tropical–subtropical species | 1 |
| Nomozaki-Akase |  | Sacoglossa |  | Plakobranchoidea | Plakobranchidae | *Thuridilla splendens* (Baba, 1949) | Tropical–subtropical species | 1 |
| Nomozaki-Akase |  | Nudipleura | Doridida | Chromodoridoidea | Chromodorididae | *Doriprismatica atromarginata* (Cuvier, 1804) | Tropical–subtropical–temperate species | 1 |
| Nomozaki-Akase |  | Nudipleura | Doridida | Chromodoridoidea | Chromodorididae | *Goniobranchus fidelis* (Kelaart, 1858) | Tropical–subtropical–temperate species | 1 |
| Nomozaki-Akase |  | Nudipleura | Doridida | Chromodoridoidea | Chromodorididae | *Chromodoris orientalis* Rudman, 1983 | Tropical–subtropical species | 5 |
| Nomozaki-Akase |  | Nudipleura | Doridida | Chromodoridoidea | Chromodorididae | *Goniobranchus sinensis* (Rudman, 1985) | Tropical–subtropical species | 3 |
| Nomozaki-Akase |  | Nudipleura | Doridida | Chromodoridoidea | Chromodorididae | *Hypselodoris festiva* (A.Adams, 1861) | Temperate species | 3 |
| Nomozaki-Akase |  | Nudipleura | Doridida | Chromodoridoidea | Chromodorididae | *Hypselodoris sagamiensis* (Baba, 1949) | Tropical–subtropical species | 1 |
| Nomozaki-Akase |  | Nudipleura | Nudibranchia | Tritonioidea | Tritoniidae | *Tritoniopsis elegans* (Audouin, 1826) | Tropical–subtropical species | 1 |
| Nomozaki-Akase | 2023-08-02 |  | Aplysiida | Aplysioidea | Aplysiidae | *Aplysia japonica* G. B. Sowerby I, 1869 | Temperate species | 2 |
| Nomozaki-Akase |  |  | Aplysiida | Aplysioidea | Aplysiidae | *Aplysia kurodai* (Baba, 1937) | Tropical–subtropical species | 3 |
| Nomozaki-Akase |  | Sacoglossa |  | Chromodoridoidea | Plakobranchidae | *Elysia trisinuata* Baba, 1949 | Tropical–subtropical species | 1 |
| Nomozaki-Akase |  | Nudipleura | Doridida | Chromodoridoidea | Chromodorididae | *Doriprismatica atromarginata* (Cuvier, 1804) | Tropical–subtropical–temperate species | 2 |
| Nomozaki-Akase |  | Nudipleura | Doridida | Chromodoridoidea | Chromodorididae | *Goniobranchus aureopurpureus* (Collingwood, 1881) | Tropical–subtropical species | 2 |
| Nomozaki-Akase |  | Nudipleura | Doridida | Chromodoridoidea | Chromodorididae | *Verconia nivalis* (Baba, 1937) | Temperate species | 1 |
| Nomozaki-Akase |  | Nudipleura | Doridida | Chromodoridoidea | Chromodorididae | *Goniobranchus sinensis* (Rudman, 1985) | Tropical–subtropical species | 1 |
| Nomozaki-Akase |  | Nudipleura | Doridida | Chromodoridoidea | Chromodorididae | *Hypselodoris festiva* (A.Adams, 1861) | Temperate species | 1 |
| Nomozaki-Akase |  | Nudipleura | Doridida | Phyllidioidea | Dendrodorididae | *Dendrodoris krusensternii* (J. E. Gray, 1850) | Tropical–subtropical–temperate species | 1 |
| Nomozaki-Akase |  | Nudipleura | Nudibranchia | Tritonioidea | Tritoniidae | *Tritoniopsis elegans* (Audouin, 1826) | Tropical–subtropical species | 3 |
| Nomozaki-Akase | 2023-09-29 | Nudipleura | Doridida | Chromodoridoidea | Chromodorididae | *Goniobranchus tinctorius* (Rüppell & Leuckart, 1830) | Tropical–subtropical species | 1 |
| Nomozaki-Akase |  | Nudipleura | Nudibranchia | Aeolidioidea | Myrrhinidae | *Phyllodesmium magnum* Rudman, 1991 | Tropical–subtropical–temperate species | 1 |
| Nomozaki-Akase |  | Nudipleura | Nudibranchia | Tritonioidea | Tritoniidae | *Tritoniopsis elegans* (Audouin, 1826) | Tropical–subtropical species | 1 |
| Nomozaki-Akase | 2023-10-03 | Sacoglossa |  | Plakobranchoidea | Plakobranchidae | *Thuridilla splendens* (Baba, 1949) | Tropical–subtropical species | 1 |
| Nomozaki-Akase |  | Nudipleura | Doridida | Chromodoridoidea | Chromodorididae | *Doriprismatica atromarginata* (Cuvier, 1804) | Tropical–subtropical–temperate species | 2 |
| Nomozaki-Akase |  | Nudipleura | Doridida | Phyllidioidea | Dendrodorididae | *Dendrodoris krusensternii* (J. E. Gray, 1850) | Tropical–subtropical–temperate species | 1 |
| Nomozaki-Akase |  | Nudipleura | Nudibranchia | Dendronotoidea | Scyllaeidae | *Scyllaea pelagica* Linnaeus, 1758 | Tropical–subtropical species | 1 |
| Nomozaki-Akase |  | Nudipleura | Nudibranchia | Samloidea | Samlidae | *Samla takashigei* Korshunova, Martynov, Bakken, Evertsen, Fletcher, Mudianta, H. Saito, Lundin, Schrödl & Picton, 2017 | Tropical–subtropical species | 4 |
| Nomozaki-Akase | 2023-11-16 | Sacoglossa |  | Plakobranchoidea | Plakobranchidae | *Thuridilla splendens* (Baba, 1949) | Tropical–subtropical species | 1 |
| Nomozaki-Akase |  | Nudipleura | Doridida | Chromodoridoidea | Chromodorididae | *Doriprismatica atromarginata* (Cuvier, 1804) | Tropical–subtropical–temperate species | 1 |
| Nomozaki-Akase |  | Nudipleura | Doridida | Chromodoridoidea | Chromodorididae | *Goniobranchus tinctorius* (Rüppell & Leuckart, 1830) | Tropical–subtropical species | 1 |
| Nomozaki-Akase |  | Nudipleura | Doridida | Chromodoridoidea | Chromodorididae | *Chromodoris orientalis* Rudman, 1983 | Tropical–subtropical species | 2 |
| Nomozaki-Akase |  | Nudipleura | Doridida | Phyllidioidea | Dendrodorididae | *Dendrodoris krusensternii* (J. E. Gray, 1850) | Tropical–subtropical–temperate species | 1 |
| Nomozaki-Akase |  | Nudipleura | Doridida | Polyceroidea | Polyceridae | *Polycera japonica* Baba, 1949 | Tropical–subtropical–temperate species | 1 |
| Nomozaki-Akase |  | Nudipleura | Nudibranchia | Aeolidioidea | Facelinidae | *Phidiana anulifera* (Baba, 1949) | Tropical–subtropical species | 1 |
| Nomozaki-Akase |  | Nudipleura | Nudibranchia | Samloidea | Samlidae | *Samla takashigei* Korshunova, Martynov, Bakken, Evertsen, Fletcher, Mudianta, H. Saito, Lundin, Schrödl & Picton, 2017 | Tropical–subtropical species | 4 |
| Nomozaki-Akase |  | Nudipleura | Nudibranchia | Tritonioidea | Tritoniidae | *Tritoniopsis elegans* (Audouin, 1826) | Tropical–subtropical species | 12 |
| Nomozaki-Akase | 2024-01-16 | Sacoglossa |  | Plakobranchoidea | Plakobranchidae | *Elysia asbecki* Wägele, Stemmer, Burghardt & Händeler, 2010 | Tropical–subtropical species | 1 |
| Nomozaki-Akase |  | Sacoglossa |  | Plakobranchoidea | Plakobranchidae | *Elysia atroviridis* Baba, 1955 | Temperate species | 1 |
| Nomozaki-Akase |  | Sacoglossa |  | Plakobranchoidea | Plakobranchidae | *Elysia japonica* Eliot, 1913 | Temperate species | 2 |
| Nomozaki-Akase |  | Sacoglossa |  | Plakobranchoidea | Plakobranchidae | *Elysia lobata* A. Gould, 1852 | Tropical–subtropical species | 1 |
| Nomozaki-Akase |  | Sacoglossa |  | Plakobranchoidea | Plakobranchidae | *Elysia ornata* (Swainson, 1840) | Tropical–subtropical–temperate species | 3 |
| Nomozaki-Akase |  | Sacoglossa |  | Plakobranchoidea | Plakobranchidae | *Thuridilla splendens* (Baba, 1949) | Tropical–subtropical species | 2 |
| Nomozaki-Akase |  | Sacoglossa |  | Plakobranchoidea | Plakobranchidae | *Thuridilla vataae (*Risbec, 1928) | Tropical–subtropical species | 2 |
| Nomozaki-Akase |  | Nudipleura | Doridida | Chromodoridoidea | Chromodorididae | *Goniobranchus tinctorius* (Rüppell & Leuckart, 1830) | Tropical–subtropical species | 2 |
| Nomozaki-Akase |  | Nudipleura | Doridida | Chromodoridoidea | Chromodorididae | *Chromodoris orientalis* Rudman, 1983 | Tropical–subtropical species | 2 |
| Nomozaki-Akase |  | Nudipleura | Doridida | Chromodoridoidea | Chromodorididae | *Hypselodoris festiva* (A.Adams, 1861) | Temperate species | 2 |
| Nomozaki-Akase |  | Nudipleura | Doridida | Phyllidioidea | Dendrodorididae | *Dendrodoris krusensternii* (J. E. Gray, 1850) | Tropical–subtropical–temperate species | 7 |
| Nomozaki-Akase |  | Nudipleura | Doridida | Polyceroidea | Polyceridae | *Polycera* sp.7 | Temperate species | 1 |
| Nomozaki-Akase |  | Nudipleura | Doridida | Onchidoridoidea | Goniodorididae | *Pelagella castanea* (Alder & Hancock, 1845) | Tropical–subtropical species | 1 |
| Nomozaki-Akase |  | Nudipleura | Nudibranchia | Aeolidioidea | Aeolidiidae | *Bulbaeolidia alba* (Risbec, 1928) | Tropical–subtropical species | 1 |
| Nomozaki-Akase |  | Nudipleura | Nudibranchia | Dendronotoidea | Dotidae | *Doto* sp. | Temperate species | 1 |
| Nomozaki-Akase |  | Nudipleura | Nudibranchia | Samloidea | Samlidae | *Samla takashigei* Korshunova, Martynov, Bakken, Evertsen, Fletcher, Mudianta, H. Saito, Lundin, Schrödl & Picton, 2017 | Tropical–subtropical species | 9 |
| Tatsunoguchi | 2023-06-02 |  | Cephalaspidea | Philinoidea | Aglajidae | *Chelidonura hirundinina* (Quoy&Gaimard, 1833) | Tropical–subtropical–temperate species | 2 |
| Tatsunoguchi |  | Sacoglossa |  | Plakobranchoidea | Plakobranchidae | *Elysia trisinuata* Baba, 1949 | Tropical–subtropical species | 1 |
| Tatsunoguchi |  | Nudipleura | Doridida | Chromodoridoidea | Chromodorididae | *Doriprismatica atromarginata* (Cuvier, 1804) | Tropical–subtropical–temperate species | 1 |
| Tatsunoguchi |  | Nudipleura | Doridida | Chromodoridoidea | Chromodorididae | *Chromodoris orientalis* Rudman, 1983 | Tropical–subtropical species | 3 |
| Tatsunoguchi |  | Nudipleura | Doridida | Chromodoridoidea | Chromodorididae | *Goniobranchus sinensis* (Rudman, 1985) | Tropical–subtropical species | 2 |
| Tatsunoguchi |  | Nudipleura | Doridida | Chromodoridoidea | Chromodorididae | *Hypselodoris sagamiensis* (Baba, 1949) | Tropical–subtropical species | 2 |
| Tatsunoguchi |  | Nudipleura | Doridida | Chromodoridoidea | Chromodorididae | *Verconia nivalis* (Baba, 1937) | Temperate species | 1 |
| Tatsunoguchi |  | Nudipleura | Doridida | Doridoidea | Discodorididae | *Jorunna parva* (Baba, 1938) | Tropical–subtropical species | 2 |
| Tatsunoguchi | 2023-07-31 | Sacoglossa |  | Plakobranchoidea | Limapontiidae | *Stiliger ornatus* Ehrenberg, 1828 | Tropical–subtropical species | 2 |
| Tatsunoguchi |  | Nudipleura | Doridida | Chromodoridoidea | Chromodorididae | *Doriprismatica atromarginata* (Cuvier, 1804) | Tropical–subtropical–temperate species | 9 |
| Tatsunoguchi |  | Nudipleura | Doridida | Chromodoridoidea | Chromodorididae | *Chromodoris orientalis* Rudman, 1983 | Tropical–subtropical species | 1 |
| Tatsunoguchi |  | Nudipleura | Doridida | Chromodoridoidea | Chromodorididae | *Goniobranchus sinensis* (Rudman, 1985) | Tropical–subtropical species | 2 |
| Tatsunoguchi |  | Nudipleura | Doridida | Chromodoridoidea | Chromodorididae | *Goniobranchus fidelis* (Kelaart, 1858) | Tropical–subtropical–temperate species | 4 |
| Tatsunoguchi |  | Nudipleura | Doridida | Phyllidioidea | Phyllidiidae | *Phyllidiella pustulosa* (Cuvier, 1804) | Tropical–subtropical–temperate species | 2 |
| Tatsunoguchi |  | Nudipleura | Nudibranchia | Tritonioidea | Tritoniidae | *Tritoniopsis elegans* (Audouin, 1826) | Tropical–subtropical species | 3 |
| Tatsunoguchi | 2023-08-29 | Sacoglossa |  | Plakobranchoidea | Plakobranchidae | *Elysia ornata* (Swainson, 1840) | Tropical–subtropical–temperate species | 2 |
| Tatsunoguchi |  | Nudipleura | Doridida | Chromodoridoidea | Chromodorididae | *Doriprismatica atromarginata* (Cuvier, 1804) | Tropical–subtropical–temperate species | 2 |
| Tatsunoguchi |  | Nudipleura | Doridida | Chromodoridoidea | Chromodorididae | *Goniobranchus fidelis* (Kelaart, 1858) | Tropical–subtropical–temperate species | 3 |
| Tatsunoguchi |  | Nudipleura | Doridida | Chromodoridoidea | Chromodorididae | *Chromodoris orientalis* Rudman, 1983 | Tropical–subtropical species | 2 |
| Tatsunoguchi |  | Nudipleura | Doridida | Chromodoridoidea | Chromodorididae | *Goniobranchus sinensis* (Rudman, 1985) | Tropical–subtropical species | 1 |
| Tatsunoguchi |  | Nudipleura | Doridida | Chromodoridoidea | Cadlinellidae | *Cadlinella ornatissima* (Risbec, 1928) | Tropical–subtropical species | 1 |
| Tatsunoguchi |  | Nudipleura | Doridida | Chromodoridoidea | Chromodorididae | *Verconia nivalis* (Baba, 1937) | Temperate species | 1 |
| Tatsunoguchi |  | Nudipleura | Doridida | Chromodoridoidea | Chromodorididae | *Goniobranchus tinctorius* (Rüppell & Leuckart, 1830) | Tropical–subtropical species | 1 |
| Tatsunoguchi |  | Nudipleura | Doridida | Chromodoridoidea | Chromodorididae | *Mexichromis multituberculata* (Baba, 1953) | Tropical–subtropical species | 1 |
| Tatsunoguchi |  | Nudipleura | Nudibranchia | Arminoidea | Arminidae | *Dermatobranchus primus* (Baba, 1976) | Temperate species | 1 |
| Tatsunoguchi |  | Nudipleura | Nudibranchia | Samloidea | Samlidae | *Samla takashigei* Korshunova, Martynov, Bakken, Evertsen, Fletcher, Mudianta, H. Saito, Lundin, Schrödl & Picton, 2017 | Tropical–subtropical species | 3 |
| Tatsunoguchi |  | Nudipleura | Nudibranchia | Tritonioidea | Tritoniidae | *Tritoniopsis elegans* (Audouin, 1826) | Tropical–subtropical species | 17 |
| Tatsunoguchi |  | Nudipleura | Nudibranchia | Unidentioidea | Unidentiidae | *Unidentia* sp. 2 | Tropical–subtropical species | 1 |
| Tatsunoguchi | 2023-09-19 | Sacoglossa |  | Plakobranchoidea | Plakobranchidae | *Elysia ornata* (Swainson, 1840) | Tropical–subtropical–temperate species | 2 |
| Tatsunoguchi |  | Nudipleura | Doridida | Chromodoridoidea | Chromodorididae | *Doriprismatica atromarginata* (Cuvier, 1804) | Tropical–subtropical–temperate species | 9 |
| Tatsunoguchi |  | Nudipleura | Doridida | Chromodoridoidea | Chromodorididae | *Goniobranchus fidelis* (Kelaart, 1858) | Tropical–subtropical–temperate species | 1 |
| Tatsunoguchi |  | Nudipleura | Doridida | Chromodoridoidea | Chromodorididae | *Chromodoris orientalis* Rudman, 1983 | Tropical–subtropical species | 1 |
| Tatsunoguchi |  | Nudipleura | Doridida | Chromodoridoidea | Chromodorididae | *Verconia nivalis* (Baba, 1937) | Temperate species | 1 |
| Tatsunoguchi |  | Nudipleura | Doridida | Chromodoridoidea | Cadlinellidae | *Cadlinella ornatissima* (Risbec, 1928) | Tropical–subtropical species | 1 |
| Tatsunoguchi |  | Nudipleura | Nudibranchia | Samloidea | Samlidae | *Samla takashigei* Korshunova, Martynov, Bakken, Evertsen, Fletcher, Mudianta, H. Saito, Lundin, Schrödl & Picton, 2017 | Tropical–subtropical species | 1 |
| Tatsunoguchi |  | Nudipleura | Nudibranchia | Tritonioidea | Tritoniidae | *Tritoniopsis elegans* (Audouin, 1826) | Tropical–subtropical species | 16 |
| Tatsunoguchi | 2023-10-23 | Sacoglossa |  | Plakobranchoidea | Limapontiidae | *Stiliger ornatus* Ehrenberg, 1828 | Tropical–subtropical species | 1 |
| Tatsunoguchi |  | Nudipleura | Doridida | Chromodoridoidea | Chromodorididae | *Goniobranchus geometricus* (Risbec, 1928) | Tropical–subtropical species | 1 |
| Tatsunoguchi |  | Nudipleura | Doridida | Chromodoridoidea | Chromodorididae | *Goniobranchus fidelis* (Kelaart, 1858) | Tropical–subtropical–temperate species | 1 |
| Tatsunoguchi |  | Nudipleura | Doridida | Chromodoridoidea | Chromodorididae | *Goniobranchus sinensis* (Rudman, 1985) | Tropical–subtropical species | 2 |
| Tatsunoguchi |  | Nudipleura | Doridida | Chromodoridoidea | Chromodorididae | *Goniobranchus tinctorius* (Rüppell & Leuckart, 1830) | Tropical–subtropical species | 2 |
| Tatsunoguchi |  | Nudipleura | Nudibranchia | Arminoidea | Arminidae | *Dermatobranchus primus* (Baba, 1976) | Temperate species | 4 |
| Tatsunoguchi |  | Nudipleura | Nudibranchia | Tritonioidea | Tritoniidae | *Tritoniopsis elegans* (Audouin, 1826) | Tropical–subtropical species | 2 |
| Tatsunoguchi | 2023-11-24 | Nudipleura | Doridida | Chromodoridoidea | Chromodorididae | *Goniobranchus tinctorius* (Rüppell & Leuckart, 1830) | Tropical–subtropical species | 1 |
| Tatsunoguchi | 2024-01-19 | Sacoglossa |  | Plakobranchoidea | Plakobranchidae | *Elysia japonica* Eliot, 1913 | Temperate species | 4 |
| Tatsunoguchi |  | Sacoglossa |  | Plakobranchoidea | Plakobranchidae | *Elysia ornata* (Swainson, 1840) | Tropical–subtropical–temperate species | 1 |
| Tatsunoguchi |  | Sacoglossa |  | Plakobranchoidea | Plakobranchidae | *Thuridilla splendens* (Baba, 1949) | Tropical–subtropical species | 1 |
| Tatsunoguchi |  | Nudipleura | Doridida | Chromodoridoidea | Chromodorididae | *Doriprismatica atromarginata* (Cuvier, 1804) | Tropical–subtropical–temperate species | 2 |
| Tatsunoguchi |  | Nudipleura | Doridida | Chromodoridoidea | Chromodorididae | *Goniobranchus tinctorius* (Rüppell & Leuckart, 1830) | Tropical–subtropical species | 3 |
| Tatsunoguchi |  | Nudipleura | Doridida | Chromodoridoidea | Chromodorididae | *Goniobranchus fidelis* (Kelaart, 1858) | Tropical–subtropical–temperate species | 1 |
| Tatsunoguchi |  | Nudipleura | Doridida | Chromodoridoidea | Chromodorididae | *Chromodoris orientalis* Rudman, 1983 | Tropical–subtropical species | 11 |
| Tatsunoguchi |  | Nudipleura | Doridida | Chromodoridoidea | Chromodorididae | *Hypselodoris sagamiensis* (Baba, 1949) | Tropical–subtropical species | 2 |
| Tatsunoguchi |  | Nudipleura | Doridida | Chromodoridoidea | Chromodorididae | *Verconia nivalis* (Baba, 1937) | Temperate species | 4 |
| Tatsunoguchi |  | Nudipleura | Doridida | Chromodoridoidea | Chromodorididae | *Verconia purpurea* (Baba, 1949) | Temperate species | 2 |
| Tatsunoguchi |  | Nudipleura | Doridida | Doridoidea | Discodorididae | *Jorunna parva* (Baba, 1938) | Tropical–subtropical species | 2 |
| Tatsunoguchi |  | Nudipleura | Doridida | Onchidoridoidea | Calycidorididae | *Diaphorodoris mitsuii* (Baba, 1938) | Tropical–subtropical species | 4 |
| Tatsunoguchi |  | Nudipleura | Doridida | Onchidoridoidea | Goniodorididae | *Bermudella japonica* (Baba, 1949) | Temperate species | 3 |
| Tatsunoguchi |  | Nudipleura | Doridida | Phyllidioidea | Dendrodorididae | *Dendrodoris krusensternii* (J. E. Gray, 1850) | Tropical–subtropical–temperate species | 4 |
| Tatsunoguchi |  | Nudipleura | Nudibranchia | Aeolidioidea | Aeolidiidae | *Bulbaeolidia alba* (Risbec, 1928) | Tropical–subtropical species | 1 |
| Tatsunoguchi |  | Nudipleura | Nudibranchia | Aeolidioidea | Facelinidae | *Caloria indica* (Bergh, 1896) | Tropical–subtropical–temperate species | 1 |
| Tatsunoguchi |  | Nudipleura | Nudibranchia | Arminoidea | Arminidae | *Dermatobranchus primus* (Baba, 1976) | Temperate species | 7 |
| Tatsunoguchi |  | Nudipleura | Nudibranchia | Fionoidea | Trinchesiidae | *Tenellia* sp.44 | Tropical–subtropical species | 1 |
| Tatsunoguchi |  | Nudipleura | Nudibranchia | Tritonioidea | Tritoniidae | *Tritoniopsis elegans* (Audouin, 1826) | Tropical–subtropical species | 2 |
| Tatsunoguchi |  | Nudipleura | Nudibranchia | Tritonioidea | Trioniidae | *Marionia* sp.1 | Temperate species | 1 |

| **Site** | **Date** | **Superorder** | **Order** | **Superfamily** | **Family** | **Species** | **Climate classification** | **Number of individuals** |
| --- | --- | --- | --- | --- | --- | --- | --- | --- |
| Nomozaki-Akase | 2023-07-05 |  | Aplysiida | Aplysioidea | Aplysiidae | *Aplysia japonica* G. B. Sowerby I, 1869 | Temperate species | 3 |
| Nomozaki-Akase |  | Sacoglossa |  | Plakobranchoidea | Plakobranchidae | *Elysia trisinuata* Baba, 1949 | Tropical–subtropical species | 3 |
| Nomozaki-Akase |  | Nudipleura | Doridida | Chromodoridoidea | Chromodorididae | *Doriprismatica* atromarginata (Cuvier, 1804) | Tropical–subtropical–temperate species | 2 |
| Nomozaki-Akase |  | Nudipleura | Doridida | Chromodoridoidea | Chromodorididae | *Chromodoris orientalis* Rudman, 1983 | Tropical–subtropical species | 2 |
| Nomozaki-Akase |  | Nudipleura | Doridida | Chromodoridoidea | Chromodorididae | *Goniobranchus sinensis* (Rudman, 1985) | Tropical–subtropical species | 1 |
| Nomozaki-Akase |  | Nudipleura | Doridida | Chromodoridoidea | Chromodorididae | *Goniobranchus tinctorius* (Rüppell & Leuckart, 1830) | Tropical–subtropical species | 2 |
| Nomozaki-Akase |  | Nudipleura | Doridida | Chromodoridoidea | Chromodorididae | *Hypselodoris festiva* (A.Adams, 1861) | Temperate species | 2 |
| Nomozaki-Akase |  | Nudipleura | Doridida | Chromodoridoidea | Chromodorididae | *Hypselodoris placida* (Baba, 1949) | Temperate species | 1 |
| Nomozaki-Akase |  | Nudipleura | Doridida | Phyllidioidea | Dendrodorididae | *Dendrodoris krusensternii* (J. E. Gray, 1850) | Tropical–subtropical–temperate species | 1 |
| Nomozaki-Akase |  | Nudipleura | Nudibranchia | Tritonioidea | Tritoniidae | *Tritoniopsis elegans* (Audouin, 1826) | Tropical–subtropical species | 2 |
| Nomozaki-Akase | 2023-07-19 |  | Aplysiida | Aplysioidea | Aplysiidae | *Aplysia japonica* G. B. Sowerby I, 1869 | Temperate species | 3 |
| Nomozaki-Akase |  | Sacoglossa |  | Plakobranchoidea | Plakobranchidae | *Elysia trisinuata* Baba, 1949 | Tropical–subtropical species | 1 |
| Nomozaki-Akase |  | Sacoglossa |  | Plakobranchoidea | Plakobranchidae | *Thuridilla* albopustulosa Gosliner, 1995 | Tropical–subtropical species | 1 |
| Nomozaki-Akase |  | Sacoglossa |  | Plakobranchoidea | Plakobranchidae | *Thuridilla splendens* (Baba, 1949) | Tropical–subtropical species | 1 |
| Nomozaki-Akase |  | Nudipleura | Doridida | Chromodoridoidea | Chromodorididae | *Doriprismatica* atromarginata (Cuvier, 1804) | Tropical–subtropical–temperate species | 1 |
| Nomozaki-Akase |  | Nudipleura | Doridida | Chromodoridoidea | Chromodorididae | *Goniobranchus fidelis* (Kelaart, 1858) | Tropical–subtropical–temperate species | 1 |
| Nomozaki-Akase |  | Nudipleura | Doridida | Chromodoridoidea | Chromodorididae | *Chromodoris orientalis* Rudman, 1983 | Tropical–subtropical species | 5 |
| Nomozaki-Akase |  | Nudipleura | Doridida | Chromodoridoidea | Chromodorididae | *Goniobranchus sinensis* (Rudman, 1985) | Tropical–subtropical species | 3 |
| Nomozaki-Akase |  | Nudipleura | Doridida | Chromodoridoidea | Chromodorididae | *Hypselodoris festiva* (A.Adams, 1861) | Temperate species | 3 |
| Nomozaki-Akase |  | Nudipleura | Doridida | Chromodoridoidea | Chromodorididae | *Hypselodoris sagamiensis* (Baba, 1949) | Tropical–subtropical species | 1 |
| Nomozaki-Akase |  | Nudipleura | Nudibranchia | Tritonioidea | Tritoniidae | *Tritoniopsis elegans* (Audouin, 1826) | Tropical–subtropical species | 1 |
| Nomozaki-Akase | 2023-08-02 |  | Aplysiida | Aplysioidea | Aplysiidae | *Aplysia japonica* G. B. Sowerby I, 1869 | Temperate species | 2 |
| Nomozaki-Akase |  |  | Aplysiida | Aplysioidea | Aplysiidae | *Aplysia kurodai* (Baba, 1937) | Tropical–subtropical species | 3 |
| Nomozaki-Akase |  | Sacoglossa |  | Chromodoridoidea | Plakobranchidae | *Elysia trisinuata* Baba, 1949 | Tropical–subtropical species | 1 |
| Nomozaki-Akase |  | Nudipleura | Doridida | Chromodoridoidea | Chromodorididae | *Doriprismatica* atromarginata (Cuvier, 1804) | Tropical–subtropical–temperate species | 2 |
| Nomozaki-Akase |  | Nudipleura | Doridida | Chromodoridoidea | Chromodorididae | *Goniobranchus aureopurpureus*(Collingwood, 1881) | Tropical–subtropical species | 2 |
| Nomozaki-Akase |  | Nudipleura | Doridida | Chromodoridoidea | Chromodorididae | *Verconia nivalis* (Baba, 1937) | Temperate species | 1 |
| Nomozaki-Akase |  | Nudipleura | Doridida | Chromodoridoidea | Chromodorididae | *Goniobranchus sinensis* (Rudman, 1985) | Tropical–subtropical species | 1 |
| Nomozaki-Akase |  | Nudipleura | Doridida | Chromodoridoidea | Chromodorididae | *Hypselodoris festiva* (A.Adams, 1861) | Temperate species | 1 |
| Nomozaki-Akase |  | Nudipleura | Doridida | Phyllidioidea | Dendrodorididae | *Dendrodoris krusensternii* (J. E. Gray, 1850) | Tropical–subtropical–temperate species | 1 |
| Nomozaki-Akase |  | Nudipleura | Nudibranchia | Tritonioidea | Tritoniidae | *Tritoniopsis elegans* (Audouin, 1826) | Tropical–subtropical species | 3 |
| Nomozaki-Akase | 2023-09-29 | Nudipleura | Doridida | Chromodoridoidea | Chromodorididae | *Goniobranchus tinctorius* (Rüppell & Leuckart, 1830) | Tropical–subtropical species | 1 |
| Nomozaki-Akase |  | Nudipleura | Nudibranchia | Aeolidioidea | Myrrhinidae | *Phyllodesmium magnum* Rudman, 1991 | Tropical–subtropical–temperate species | 1 |
| Nomozaki-Akase |  | Nudipleura | Nudibranchia | Tritonioidea | Tritoniidae | *Tritoniopsis elegans* (Audouin, 1826) | Tropical–subtropical species | 1 |
| Nomozaki-Akase | 2023-10-03 | Sacoglossa |  | Plakobranchoidea | Plakobranchidae | *Thuridilla splendens* (Baba, 1949) | Tropical–subtropical species | 1 |
| Nomozaki-Akase |  | Nudipleura | Doridida | Chromodoridoidea | Chromodorididae | *Doriprismatica* atromarginata (Cuvier, 1804) | Tropical–subtropical–temperate species | 2 |
| Nomozaki-Akase |  | Nudipleura | Doridida | Phyllidioidea | Dendrodorididae | *Dendrodoris krusensternii* (J. E. Gray, 1850) | Tropical–subtropical–temperate species | 1 |
| Nomozaki-Akase |  | Nudipleura | Nudibranchia | Dendronotoidea | Scyllaeidae | *Scyllaea pelagica Linnaeus*, 1758 | Tropical–subtropical species | 1 |
| Nomozaki-Akase |  | Nudipleura | Nudibranchia | Samloidea | Samlidae | *Samla takashigei* Korshunova, Martynov, Bakken, Evertsen, Fletcher, Mudianta, H. Saito, Lundin, Schrödl & Picton, 2017 | Tropical–subtropical species | 4 |
| Nomozaki-Akase | 2023-11-16 | Sacoglossa |  | Plakobranchoidea | Plakobranchidae | *Thuridilla splendens* (Baba, 1949) | Tropical–subtropical species | 1 |
| Nomozaki-Akase |  | Nudipleura | Doridida | Chromodoridoidea | Chromodorididae | *Doriprismatica* atromarginata (Cuvier, 1804) | Tropical–subtropical–temperate species | 1 |
| Nomozaki-Akase |  | Nudipleura | Doridida | Chromodoridoidea | Chromodorididae | *Goniobranchus tinctorius* (Rüppell & Leuckart, 1830) | Tropical–subtropical species | 1 |
| Nomozaki-Akase |  | Nudipleura | Doridida | Chromodoridoidea | Chromodorididae | *Chromodoris orientalis* Rudman, 1983 | Tropical–subtropical species | 2 |
| Nomozaki-Akase |  | Nudipleura | Doridida | Phyllidioidea | Dendrodorididae | *Dendrodoris krusensternii* (J. E. Gray, 1850) | Tropical–subtropical–temperate species | 1 |
| Nomozaki-Akase |  | Nudipleura | Doridida | Polyceroidea | Polyceridae | *Polycera japonica* Baba, 1949 | Tropical–subtropical–temperate species | 1 |
| Nomozaki-Akase |  | Nudipleura | Nudibranchia | Aeolidioidea | Facelinidae | *Phidiana anulifera* (Baba, 1949) | Tropical–subtropical species | 1 |
| Nomozaki-Akase |  | Nudipleura | Nudibranchia | Samloidea | Samlidae | *Samla takashigei* Korshunova, Martynov, Bakken, Evertsen, Fletcher, Mudianta, H. Saito, Lundin, Schrödl & Picton, 2017 | Tropical–subtropical species | 4 |
| Nomozaki-Akase |  | Nudipleura | Nudibranchia | Tritonioidea | Tritoniidae | *Tritoniopsis elegans* (Audouin, 1826) | Tropical–subtropical species | 12 |
| Nomozaki-Akase | 2024-01-16 | Sacoglossa |  | Plakobranchoidea | Plakobranchidae | *Elysia asbecki* Wägele, Stemmer, Burghardt & Händeler, 2010 | Tropical–subtropical species | 1 |
| Nomozaki-Akase |  | Sacoglossa |  | Plakobranchoidea | Plakobranchidae | *Elysia atroviridis* Baba, 1955 | Temperate species | 1 |
| Nomozaki-Akase |  | Sacoglossa |  | Plakobranchoidea | Plakobranchidae | *Elysia japonica* Eliot, 1913 | Temperate species | 2 |
| Nomozaki-Akase |  | Sacoglossa |  | Plakobranchoidea | Plakobranchidae | *Elysia lobata* A. Gould, 1852 | Tropical–subtropical species | 1 |
| Nomozaki-Akase |  | Sacoglossa |  | Plakobranchoidea | Plakobranchidae | *Elysia ornata* (Swainson, 1840) | Tropical–subtropical–temperate species | 3 |
| Nomozaki-Akase |  | Sacoglossa |  | Plakobranchoidea | Plakobranchidae | *Thuridilla splendens* (Baba, 1949) | Tropical–subtropical species | 2 |
| Nomozaki-Akase |  | Sacoglossa |  | Plakobranchoidea | Plakobranchidae | *Thuridilla vataae (*Risbec, 1928) | Tropical–subtropical species | 2 |
| Nomozaki-Akase |  | Nudipleura | Doridida | Chromodoridoidea | Chromodorididae | *Goniobranchus tinctorius* (Rüppell & Leuckart, 1830) | Tropical–subtropical species | 2 |
| Nomozaki-Akase |  | Nudipleura | Doridida | Chromodoridoidea | Chromodorididae | *Chromodoris orientalis* Rudman, 1983 | Tropical–subtropical species | 2 |
| Nomozaki-Akase |  | Nudipleura | Doridida | Chromodoridoidea | Chromodorididae | *Hypselodoris festiva* (A.Adams, 1861) | Temperate species | 2 |
| Nomozaki-Akase |  | Nudipleura | Doridida | Phyllidioidea | Dendrodorididae | *Dendrodoris krusensternii* (J. E. Gray, 1850) | Tropical–subtropical–temperate species | 7 |
| Nomozaki-Akase |  | Nudipleura | Doridida | Polyceroidea | Polyceridae | *Polycera* sp.7 | Temperate species | 1 |
| Nomozaki-Akase |  | Nudipleura | Doridida | Onchidoridoidea | Goniodorididae | *Pelagella castanea* (Alder & Hancock, 1845) | Tropical–subtropical species | 1 |
| Nomozaki-Akase |  | Nudipleura | Nudibranchia | Aeolidioidea | Aeolidiidae | *Bulbaeolidia alba* (Risbec, 1928) | Tropical–subtropical species | 1 |
| Nomozaki-Akase |  | Nudipleura | Nudibranchia | Dendronotoidea | Dotidae | *Doto* sp. | Temperate species | 1 |
| Nomozaki-Akase |  | Nudipleura | Nudibranchia | Samloidea | Samlidae | *Samla takashigei* Korshunova, Martynov, Bakken, Evertsen, Fletcher, Mudianta, H. Saito, Lundin, Schrödl & Picton, 2017 | Tropical–subtropical species | 9 |
| Tatsunoguchi | 2023-06-02 |  | Cephalaspidea | Philinoidea | Aglajidae | *Chelidonura hirundinina* (Quoy & Gaimard, 1833) | Tropical–subtropical–temperate species | 2 |
| Tatsunoguchi |  | Sacoglossa |  | Plakobranchoidea | Plakobranchidae | *Elysia trisinuata* Baba, 1949 | Tropical–subtropical species | 1 |
| Tatsunoguchi |  | Nudipleura | Doridida | Chromodoridoidea | Chromodorididae | *Doriprismatica* atromarginata (Cuvier, 1804) | Tropical–subtropical–temperate species | 1 |
| Tatsunoguchi |  | Nudipleura | Doridida | Chromodoridoidea | Chromodorididae | *Chromodoris orientalis* Rudman, 1983 | Tropical–subtropical species | 3 |
| Tatsunoguchi |  | Nudipleura | Doridida | Chromodoridoidea | Chromodorididae | *Goniobranchus sinensis* (Rudman, 1985) | Tropical–subtropical species | 2 |
| Tatsunoguchi |  | Nudipleura | Doridida | Chromodoridoidea | Chromodorididae | *Hypselodoris sagamiensis* (Baba, 1949) | Tropical–subtropical species | 2 |
| Tatsunoguchi |  | Nudipleura | Doridida | Chromodoridoidea | Chromodorididae | *Verconia nivalis* (Baba, 1937) | Temperate species | 1 |
| Tatsunoguchi |  | Nudipleura | Doridida | Doridoidea | Discodorididae | *Jorunna parva*(Baba, 1938) | Tropical–subtropical species | 2 |
| Tatsunoguchi | 2023-07-31 | Sacoglossa |  | Plakobranchoidea | Limapontiidae | *Stiliger ornatus* Ehrenberg, 1828 | Tropical–subtropical species | 2 |
| Tatsunoguchi |  | Nudipleura | Doridida | Chromodoridoidea | Chromodorididae | *Doriprismatica* atromarginata (Cuvier, 1804) | Tropical–subtropical–temperate species | 9 |
| Tatsunoguchi |  | Nudipleura | Doridida | Chromodoridoidea | Chromodorididae | *Chromodoris orientalis* Rudman, 1983 | Tropical–subtropical species | 1 |
| Tatsunoguchi |  | Nudipleura | Doridida | Chromodoridoidea | Chromodorididae | *Goniobranchus sinensis* (Rudman, 1985) | Tropical–subtropical species | 2 |
| Tatsunoguchi |  | Nudipleura | Doridida | Chromodoridoidea | Chromodorididae | *Goniobranchus fidelis* (Kelaart, 1858) | Tropical–subtropical–temperate species | 4 |
| Tatsunoguchi |  | Nudipleura | Doridida | Phyllidioidea | Phyllidiidae | *Phyllidiella pustulosa* (Cuvier, 1804) | Tropical–subtropical–temperate species | 2 |
| Tatsunoguchi |  | Nudipleura | Nudibranchia | Tritonioidea | Tritoniidae | *Tritoniopsis elegans* (Audouin, 1826) | Tropical–subtropical species | 3 |
| Tatsunoguchi | 2023-08-29 | Sacoglossa |  | Plakobranchoidea | Plakobranchidae | *Elysia ornata* (Swainson, 1840) | Tropical–subtropical–temperate species | 2 |
| Tatsunoguchi |  | Nudipleura | Doridida | Chromodoridoidea | Chromodorididae | *Doriprismatica* atromarginata (Cuvier, 1804) | Tropical–subtropical–temperate species | 2 |
| Tatsunoguchi |  | Nudipleura | Doridida | Chromodoridoidea | Chromodorididae | *Goniobranchus fidelis* (Kelaart, 1858) | Tropical–subtropical–temperate species | 3 |
| Tatsunoguchi |  | Nudipleura | Doridida | Chromodoridoidea | Chromodorididae | *Chromodoris orientalis* Rudman, 1983 | Tropical–subtropical species | 2 |
| Tatsunoguchi |  | Nudipleura | Doridida | Chromodoridoidea | Chromodorididae | *Goniobranchus sinensis* (Rudman, 1985) | Tropical–subtropical species | 1 |
| Tatsunoguchi |  | Nudipleura | Doridida | Chromodoridoidea | Cadlinellidae | *Cadlinella ornatissima* (Risbec, 1928) | Tropical–subtropical species | 1 |
| Tatsunoguchi |  | Nudipleura | Doridida | Chromodoridoidea | Chromodorididae | *Verconia nivalis* (Baba, 1937) | Temperate species | 1 |
| Tatsunoguchi |  | Nudipleura | Doridida | Chromodoridoidea | Chromodorididae | *Goniobranchus tinctorius* (Rüppell & Leuckart, 1830) | Tropical–subtropical species | 1 |
| Tatsunoguchi |  | Nudipleura | Doridida | Chromodoridoidea | Chromodorididae | *Mexichromis multituberculata* (Baba, 1953) | Tropical–subtropical species | 1 |
| Tatsunoguchi |  | Nudipleura | Nudibranchia | Arminoidea | Arminidae | *Dermatobranchus primus* (Baba, 1976) | Temperate species | 1 |
| Tatsunoguchi |  | Nudipleura | Nudibranchia | Samloidea | Samlidae | *Samla takashigei* Korshunova, Martynov, Bakken, Evertsen, Fletcher, Mudianta, H. Saito, Lundin, Schrödl & Picton, 2017 | Tropical–subtropical species | 3 |
| Tatsunoguchi |  | Nudipleura | Nudibranchia | Tritonioidea | Tritoniidae | *Tritoniopsis elegans* (Audouin, 1826) | Tropical–subtropical species | 17 |
| Tatsunoguchi |  | Nudipleura | Nudibranchia | Unidentioidea | Unidentiidae | *Unidentia* sp. 2 | Tropical–subtropical species | 1 |
| Tatsunoguchi | 2023-09-19 | Sacoglossa |  | Plakobranchoidea | Plakobranchidae | *Elysia ornata* (Swainson, 1840) | Tropical–subtropical–temperate species | 2 |
| Tatsunoguchi |  | Nudipleura | Doridida | Chromodoridoidea | Chromodorididae | *Doriprismatica* atromarginata (Cuvier, 1804) | Tropical–subtropical–temperate species | 9 |
| Tatsunoguchi |  | Nudipleura | Doridida | Chromodoridoidea | Chromodorididae | *Goniobranchus fidelis* (Kelaart, 1858) | Tropical–subtropical–temperate species | 1 |
| Tatsunoguchi |  | Nudipleura | Doridida | Chromodoridoidea | Chromodorididae | *Chromodoris orientalis* Rudman, 1983 | Tropical–subtropical species | 1 |
| Tatsunoguchi |  | Nudipleura | Doridida | Chromodoridoidea | Chromodorididae | *Verconia nivalis* (Baba, 1937) | Temperate species | 1 |
| Tatsunoguchi |  | Nudipleura | Doridida | Chromodoridoidea | Cadlinellidae | *Cadlinella ornatissima* (Risbec, 1928) | Tropical–subtropical species | 1 |
| Tatsunoguchi |  | Nudipleura | Nudibranchia | Samloidea | Samlidae | *Samla takashigei* Korshunova, Martynov, Bakken, Evertsen, Fletcher, Mudianta, H. Saito, Lundin, Schrödl & Picton, 2017 | Tropical–subtropical species | 1 |
| Tatsunoguchi |  | Nudipleura | Nudibranchia | Tritonioidea | Tritoniidae | *Tritoniopsis elegans* (Audouin, 1826) | Tropical–subtropical species | 16 |
| Tatsunoguchi | 2023-10-23 | Sacoglossa |  | Plakobranchoidea | Limapontiidae | *Stiliger ornatus* Ehrenberg, 1828 | Tropical–subtropical species | 1 |
| Tatsunoguchi |  | Nudipleura | Doridida | Chromodoridoidea | Chromodorididae | *Goniobranchus geometricus* (Risbec, 1928) | Tropical–subtropical species | 1 |
| Tatsunoguchi |  | Nudipleura | Doridida | Chromodoridoidea | Chromodorididae | *Goniobranchus fidelis* (Kelaart, 1858) | Tropical–subtropical–temperate species | 1 |
| Tatsunoguchi |  | Nudipleura | Doridida | Chromodoridoidea | Chromodorididae | *Goniobranchus sinensis* (Rudman, 1985) | Tropical–subtropical species | 2 |
| Tatsunoguchi |  | Nudipleura | Doridida | Chromodoridoidea | Chromodorididae | *Goniobranchus tinctorius* (Rüppell & Leuckart, 1830) | Tropical–subtropical species | 2 |
| Tatsunoguchi |  | Nudipleura | Nudibranchia | Arminoidea | Arminidae | *Dermatobranchus primus* (Baba, 1976) | Temperate species | 4 |
| Tatsunoguchi |  | Nudipleura | Nudibranchia | Tritonioidea | Tritoniidae | *Tritoniopsis elegans* (Audouin, 1826) | Tropical–subtropical species | 2 |
| Tatsunoguchi | 2023-11-24 | Nudipleura | Doridida | Chromodoridoidea | Chromodorididae | *Goniobranchus tinctorius* (Rüppell & Leuckart, 1830) | Tropical–subtropical species | 1 |
| Tatsunoguchi | 2024-01-19 | Sacoglossa |  | Plakobranchoidea | Plakobranchidae | *Elysia japonica* Eliot, 1913 | Temperate species | 4 |
| Tatsunoguchi |  | Sacoglossa |  | Plakobranchoidea | Plakobranchidae | *Elysia ornata* (Swainson, 1840) | Tropical–subtropical–temperate species | 1 |
| Tatsunoguchi |  | Sacoglossa |  | Plakobranchoidea | Plakobranchidae | *Thuridilla splendens* (Baba, 1949) | Tropical–subtropical species | 1 |
| Tatsunoguchi |  | Nudipleura | Doridida | Chromodoridoidea | Chromodorididae | *Doriprismatica* atromarginata (Cuvier, 1804) | Tropical–subtropical–temperate species | 2 |
| Tatsunoguchi |  | Nudipleura | Doridida | Chromodoridoidea | Chromodorididae | *Goniobranchus tinctorius* (Rüppell & Leuckart, 1830) | Tropical–subtropical species | 3 |
| Tatsunoguchi |  | Nudipleura | Doridida | Chromodoridoidea | Chromodorididae | *Goniobranchus fidelis* (Kelaart, 1858) | Tropical–subtropical–temperate species | 1 |
| Tatsunoguchi |  | Nudipleura | Doridida | Chromodoridoidea | Chromodorididae | *Chromodoris orientalis* Rudman, 1983 | Tropical–subtropical species | 11 |
| Tatsunoguchi |  | Nudipleura | Doridida | Chromodoridoidea | Chromodorididae | *Hypselodoris sagamiensis* (Baba, 1949) | Tropical–subtropical species | 2 |
| Tatsunoguchi |  | Nudipleura | Doridida | Chromodoridoidea | Chromodorididae | *Verconia nivalis* (Baba, 1937) | Temperate species | 4 |
| Tatsunoguchi |  | Nudipleura | Doridida | Chromodoridoidea | Chromodorididae | *Verconia purpurea* (Baba, 1949) | Temperate species | 2 |
| Tatsunoguchi |  | Nudipleura | Doridida | Doridoidea | Discodorididae | *Jorunna parva*(Baba, 1938) | Tropical–subtropical species | 2 |
| Tatsunoguchi |  | Nudipleura | Doridida | Onchidoridoidea | Calycidorididae | *Diaphorodoris mitsuii* (Baba, 1938) | Tropical–subtropical species | 4 |
| Tatsunoguchi |  | Nudipleura | Doridida | Onchidoridoidea | Goniodorididae | *Bermudella japonica* (Baba, 1949) | Temperate species | 3 |
| Tatsunoguchi |  | Nudipleura | Doridida | Phyllidioidea | Dendrodorididae | *Dendrodoris krusensternii* (J. E. Gray, 1850) | Tropical–subtropical–temperate species | 4 |
| Tatsunoguchi |  | Nudipleura | Nudibranchia | Aeolidioidea | Aeolidiidae | *Bulbaeolidia alba* (Risbec, 1928) | Tropical–subtropical species | 1 |
| Tatsunoguchi |  | Nudipleura | Nudibranchia | Aeolidioidea | Facelinidae | *Caloria indica* (Bergh, 1896) | Tropical–subtropical–temperate species | 1 |
| Tatsunoguchi |  | Nudipleura | Nudibranchia | Arminoidea | Arminidae | *Dermatobranchus primus* (Baba, 1976) | Temperate species | 7 |
| Tatsunoguchi |  | Nudipleura | Nudibranchia | Fionoidea | Trinchesiidae | *Tenellia* sp.44 | Tropical–subtropical species | 1 |
| Tatsunoguchi |  | Nudipleura | Nudibranchia | Tritonioidea | Tritoniidae | *Tritoniopsis elegans* (Audouin, 1826) | Tropical–subtropical species | 2 |
| Tatsunoguchi |  | Nudipleura | Nudibranchia | Tritonioidea | Trioniidae | *Marionia* sp.1 | Temperate species | 1 |
